# Mind the gap! Integrating taxonomic approaches to assess ant diversity at the southern extreme of the Atlantic Forest

**DOI:** 10.1002/ece3.3549

**Published:** 2017-11-10

**Authors:** Priscila Elena Hanisch, Pablo D. Lavinia, Andrew V. Suarez, Darío Alejandro Lijtmaer, Maurice Leponce, Carolina Ivon Paris, Pablo Luis Tubaro

**Affiliations:** ^1^ Museo Argentino de Ciencias Naturales “Bernardino Rivadavia” MACN‐CONICET Buenos Aires Argentina; ^2^ Department of Entomology and Department of Animal Biology University of Illinois Urbana USA; ^3^ Aquatic and Terrestrial Ecology unit Royal Belgian Institute of Natural Sciences Brussels Belgium; ^4^ Departamento Ecología Genética y Evolución Universidad de Buenos Aires Buenos Aires Argentina

**Keywords:** Argentina, DNA barcoding, Formicidae, Iguazú National Park, species delimitation

## Abstract

Understanding patterns of species diversity relies on accurate taxonomy which can only be achieved by long‐term natural history research and the use of complementary information to establish species boundaries among cryptic taxa. We used DNA barcoding to characterize the ant diversity of Iguazú National Park (INP), a protected area of the Upper Paraná Atlantic Forest ecoregion, located at the southernmost extent of this forest. We assessed ant diversity using both cytochrome c oxidase subunit 1 (COI) sequences and traditional morphological approaches, and compared the results of these two methods. We successfully obtained COI sequences for 312 specimens belonging to 124 species, providing a DNA barcode reference library for nearly 50% of the currently known ant fauna of INP. Our results support a clear barcode gap for all but two species, with a mean intraspecific divergence of 0.72%, and an average congeneric distance of 17.25%. Congruently, the library assembled here was useful for the discrimination of the ants of INP and allowed us to link unidentified males and queens to their worker castes. To detect overlooked diversity, we classified the DNA barcodes into Molecular Operational Taxonomic Units (MOTUs) using three different clustering algorithms, and compared their number and composition to that of reference species identified based on morphology. The MOTU count was always higher than that of reference species regardless of the method, suggesting that the diversity of ants at INP could be between 6% and 10% higher than currently recognized. Lastly, our survey contributed with 78 new barcode clusters to the global DNA barcode reference library, and added 36 new records of ant species for the INP, being 23 of them new citations for Argentina.

## 1 INTRODUCTION

Comprehensive species inventories are prerequisites for conservation planning and for understanding ecological processes such as the role of biodiversity in ecosystem stability and function (Bickford et al., 2007; Coleman & Whitman, 2005; Mace, 2004). Moreover, a misinterpretation of alpha diversity can have negative impacts on human welfare. For example, misidentification of disease vectors, species subject to human consumption, and agricultural pests can result in substantial harm to economies and human health (Besansky, 1999). Nevertheless, the achievement of near complete species inventories requires methodologically diverse sampling and long‐term research (Longino, Coddington, & Colwell, 2002; Wild, 2007a). Traditionally, species identification and description rely solely on morphological characters, but with the advent of molecular tools, other approaches have become available. In particular, the use of sequence‐based specimen identification, known as DNA barcoding (Hebert, Cywinska, Ball, & deWaard, 2003), is increasingly proving to be a useful tool for species identification and diversity assessment (e.g., Delsinne et al., 2012; Ferreira, Poteaux, Delabie, Fresneau, & Rybak, 2010; Hebert, Penton, Burns, Janzen, & Hallwachs, 2004; Zenker et al., 2016). This technique is based on the amplification and analysis of a standardized short sequence of mitochondrial DNA near the 5′ end of the cytochrome *c* oxidase subunit I (COI) gene (for the majority of the animal kingdom) and relies on the premise that intraspecific diversity is predictably lower than interspecific diversity at this locus, even between closely related (i.e., sister) species (Hebert, Cywinska, et al., 2003; Hebert, Ratnasingham, & Waard, 2003).

DNA barcoding can provide a rapid and efficient way to catalog diversity before it disappears as a consequence of human activities (Floyd, Wilson, & Hebert, 2009). This is particularly true for diverse and understudied taxa in threatened habitats, such as insects in tropical forests (Myers, Mittermeier, Mittermeier, da Fonseca, & Kent, 2000). Moreover, when coupled with different clustering algorithms, DNA barcodes can be used to delimit Molecular Operational Taxonomic Units (MOTUs): clusters of sequences grouped together based on similarity (Floyd, Abebe, Papert, & Blaxter, 2002). These MOTUs can then be used to accelerate specimen identification, unveil cryptic diversity, test species delimitation hypothesis (e.g., Ramalho, Santos, Fernandes, Morini, & Bueno, 2016), or to perform fast census of animal diversity that could serve as the basis for subsequent taxonomic work (e.g., Smith, Hallwachs, Janzen, & Longino, 2014).

Ants are an ecologically dominant group of insects in most terrestrial communities, especially in tropical ecosystems where they can exceed vertebrates in biomass (Hölldobler & Wilson, 1990). They play a major role in ecosystem functioning as predators, scavengers, mutualists, and ecosystem engineers (Folgarait, 1998). As with many arthropod taxa, ants are often difficult to identify species, with highly diverse and ecologically important ant genera still lacking comprehensive identification tools (e.g., *Solenopsis*,* Pheidole*,* Camponotus*,* Hypoponera*). Moreover, ant morphology varies both among and within castes; species can have polymorphic workers or specialized reproductive forms (as in some *Hypoponera*,* Platythyrea*) (Hölldobler & Wilson, 1990; Peeters & Ito, 2001). The most common ant sampling methods often collect only workers (e.g., pitfall traps) or flying reproductives (e.g., Malaise or light traps) rather than whole colonies where different castes can be associated. Coupled with the limitation that most taxonomic keys to species level are based solely on worker castes, associating queens and males to workers in inventories can be problematic. This undermines the scope of diversity studies and ecological work in general, for example, by impeding the study of the phenology of ant reproduction (e.g., Feitosa et al., 2016; Kaspari, Pickering, & Windsor, 2001).

DNA barcodes have been used to aid in studies of ant diversity and to delimit species boundaries in taxonomically difficult groups (e.g., Ferreira et al., 2010; Schlick‐Steiner et al., 2006; Smith & Fisher, 2009; Smith et al., 2014). In addition, DNA barcode reference libraries for ants allow other objectives such as caste associations (e.g., Smith, Janzen, Hallwachs, & Longino, 2015). In this study, we generated a DNA barcode reference library for the ants of the Iguazú National Park (INP), a protected area located at the southern extreme of the Atlantic Forest, a biodiversity hotspot in eastern South America (Myers et al., 2000). This reference library included 312 specimens from 182 species, around 50% of the known ant diversity of the INP (Hanisch et al., 2015). We tested the efficacy of this DNA library by performing specimen identification simulations and used the library to identify individuals for which keys were unavailable (mostly males and queens). We also estimated the number of MOTUs using different species delineation algorithms to uncover hidden diversity not detected by morphology. Finally, we compared MOTU counts and their composition across methods and assessed the correspondence between reference species and MOTUs boundaries.

## 2 MATERIALS AND METHODS

### 2.1 Study site

Iguazú National Park is a 67,000 ha protected area situated in northwestern Misiones, Argentina (25°40′48.54″S, 54°27′15.09″W). The climate is humid subtropical with no defined dry season, and mean monthly temperatures ranging from 15°C (June–August) to 26°C (December–February). Annual rainfall ranges between 1,800 and 2,000 mm and humidity is between 70% and 90%.

### 2.2 Ant surveys

We collected ants during different collection events in 1998, 1999, 2003, 2005, 2008, 2009, and 2011 at 21 sites in INP, via light and pitfall traps, litter samples, subterranean and surface baits, and hand‐collecting events (Hanisch et al., 2015). We made additional hand sampling collection during summer of 2015 and 2016 to target other microhabitats and additional areas of INP. Altogether, this study is based on specimens from over 118 litter samples, 78 pitfall traps, 228 surface baits, 57 underground baits, and 348 hand‐collecting events. Collected ants were preserved in ethanol 96% and identified using the available literature (Boudinot, Sumnicht, & Adams, 2013; Brown, 1978; Dash, 2011; Fernandes, De Oliveira, & Delabie, 2014; Jiménez, Fernández, Arias, & Lozano‐Zambrano, 2008; Kempf, 1962, 1965; Kugler & Brown, 1982; Lattke, Fernández, & Palacio, 2007; Lenhart, Dash, & Mackay, 2013; Longino & Fernández, 2007; MacKay, 1996; Mackay, & Mackay, 2010; Ortíz Sepúlveda, 2012; Ronque, Azevedo‐Silva, Mori, Souza, & Oliveira, 2015; Wild, 2005, 2007b). If we were unable to key out specimens reliably to species, they were assigned to a morphospecies.

### 2.3 DNA extraction and amplification

Genomic DNA was obtained from a leg (or more than one in cases of very small specimens) following a glass fiber‐based extraction protocol developed by Ivanova, Dewaard, and Hebert (2006). A 658‐bp fragment near the 5′ end of the COI gene was amplified following standard protocols developed for DNA barcoding (Wilson, 2012) and using two sets of primers: LepF1 and LepR1 (Hebert et al., 2004), and the primer cocktails C_LepFolF [LepF1 + LCO1490 (Folmer, Hoeh, Black, & Vrijenhoek, 1994)] and C_LepFolR [(LepR1 + HCO2198 (Folmer et al., 1994)]. The cocktails were implemented to increase the amplification success for the oldest samples, for specimens that were not preserved under DNA‐friendly conditions (e.g., stored at room temperature), and for cases of poor primer fit. DNA extraction and COI amplification were performed at the Museo Argentino de Ciencias Naturales “Bernardino Rivadavia” (MACN), in Buenos Aires, Argentina, while sequencing was performed bidirectionally at the Canadian Centre for DNA Barcoding (CCDB; University of Guelph, Canada) with the same primers used for amplification. Residual genomic DNA was deposited, together with a tissue sample, at the National Ultrafrozen Tissue Collection at the MACN.

Sequences were edited and aligned using CodonCodeAlligner 4.0.4 (CondonCode Corporation, Dedham, MA) and translated into amino acid sequence to verify the lack of stop codons within the reading frame. Sequences were also examined to assess the presence of indels in the alignment using MEGA 5.0 (Tamura et al., 2011). All sequences obtained in this study together with their corresponding trace files, collection data, taxonomic information, and images are available on BOLD in the public dataset “DS‐AOI16ALL” (https://doi.org/10.5883/ds-aoi16all). Sequences are also deposited in GenBank (accession numbers MF925738–MF926049). All relevant information for each specimen is summarized in the Table S1.

### 2.4 Sequence analyses

#### 2.4.1 Final dataset

Only sequences belonging to identified individuals, with at least 500 bp and with less than 1% ambiguous calls were included in the genetic analyses described in the next sections. Eight records with contamination were excluded, along with 30 good‐quality sequences that were not possible to identify species with confidence (i.e., minor workers, males, and queens) as required by our analysis. However, we did use these 30 sequences to test the utility of our barcode library for species name assignment.

#### 2.4.2 Genetic distances

We compared intra‐ and interspecific genetic distances both as uncorrected divergence values (i.e., *p*‐distance) and using the Kimura 2‐parameter (K2P) distance model (Kimura, 1980). As the results were almost identical between these two methods, and because K2P is the most common model implemented in DNA barcoding and allows a more direct comparison with previous studies, we only report those obtained using K2P. Missing data were handled using the pairwise deletion approach. The mean intraspecific divergence was obtained with the package SPIDER (Brown et al., 2012) in R 3.3.1 (R Core Team, 2016) for all species represented by two or more individuals. As a measure of interspecific distance, we estimated the mean distance among congeneric species for those genera represented by at least two species using the Distance Summary tool available on BOLD. To test for the existence of a barcode gap (i.e., a separation between intra‐ and interspecific genetic variation; Meier, Zhang, Ali, & Zamudio, 2008; Meyer & Paulay, 2005), we compared for each specimen the distance to its furthest conspecific and to its closest heterospecific.

#### 2.4.3 Gene trees

We generated a neighbor‐joining (NJ) tree in BOLD using the Taxon ID tree tool (K2P and pairwise deletion were used). Node support was computed with 1,000 bootstrap pseudoreplicates performed in MEGA. Additionally, we estimated a maximum likelihood (ML) gene tree using RAxML 8.1.22 (Stamatakis, 2014). The analysis consisted of 100 independent ML tree searches and 1,000 rapid bootstrap pseudoreplicates under the GTRGAMMA model of evolution. Support values were printed on the best tree found among the ML searches. It is worth mentioning that our objective here was not to infer the phylogenetic relationships between the species analyzed but to obtain support values for terminal nodes (i.e., species or morphospecies) and intraspecific genetic clusters that may represent new, cryptic species.

### 2.5 Specimen identification simulations

To assess the utility of our COI barcode library for species name assignment, we simulated a sequence‐based identification process (Barco, Raupach, Laakmann, Neumann, & Knebelsberger, 2016). We ran each sequence in our dataset (treated as an unknown specimen for the purpose of the test) against our complete library of identified sequences in order to assign a species name to the “unknown” query. This species name was assigned based on three different criteria: Best Match (BM) and Best Close Match (BCM) as defined by Meier, Shiyang, Vaidya, Ng, and Hedin (2006), and the BOLD Identification Criterion (BIC) as implemented in the BOLD ID engine (Ratnasingham & Hebert, 2007). In the case of the first two criteria, simulations were carried out using the Species Identifier Tool of Taxon DNA 1.8 (Meier et al., 2006), while for the BIC approach, we used SPIDER. Under the BM criterion, a species name is assigned to the query sequence according to the closest match (the one with the lowest genetic distance) available at the library regardless of the divergence. The BCM criterion works like the BM, but it incorporates a threshold defined by the user in order to make the identification process more rigorous. Here, a species name is assigned only if the closest match to the query sequence has a sequence divergence below the specified distance threshold. Therefore, if a query sequence has two (or more) equally close matches of different species including at least one conspecific, the result would be ambiguous, while if the closest match corresponds to a heterospecific sequence, it would be considered an incorrect identification. If the closest match is found outside the threshold, the query remains as unidentified. Lastly, the BIC constitutes an even more strict approach as it looks at all the sequences within the threshold. When all sequences below the threshold are conspecific to the query, the identification is correct, while it is considered ambiguous when both homo‐ and heterospecific sequences are found within the threshold of the query. An incorrect identification happens when all matches below the threshold correspond to species different to that of the query, while the query remains unidentified when no match is found within the threshold.

For the BCM and the BIC criteria, we implemented four different thresholds: 1—the 95th percentile of all intraspecific distances, where the threshold corresponds to the genetic distance below which 95% of all intraspecific distances are found (Meier et al., 2006), 2—the BOLD ID engine threshold of 1% sequence divergence (Ratnasingham & Hebert, 2007), 3—the divergence value that minimizes the false‐positive and false‐negative identification errors (i.e., the cumulative error) obtained with the “thresVal” function in SPIDER, and 4—the minimum value in a density plot of all genetic distances which is commonly interpreted as the transition between intra‐ and interspecific distances, obtained with the function “localMinima” in SPIDER. Singletons were not used as queries, but they remained as potential matches for the rest of the sequences. Results were identical using K2P and uncorrected distances, so we report only the former.

In addition to the simulations described above, we queried the 30 sequences that belonged to unidentified males, queens, and minor workers against both our database (using Species Identifier Tool) and BOLD's entire library as of January 2017 (through BOLD's Identification engine) to get a species identification. We registered the closest match for each of both libraries and then compared the outcomes.

### 2.6 Assessment of cryptic diversity through MOTU delineation

We used three different distance‐based clustering methods to delimit MOTUs within our barcode database: Automatic Barcode Gap Discovery (ABGD, Puillandre, Lambert, Brouillet, & Achaz, 2012), statistical parsimony networks (Templeton, Crandall, & Sing, 1992) as implemented in TCS (Clement, Posada, & Crandall, 2000), and the Refined Single Linkage algorithm (RESL, Ratnasingham & Hebert, 2013). Briefly, these methods partition the sequences into MOTUs based on different similarity cutoffs depending on the clustering algorithm. In the case of ABGD, it is a statistical recursive method that explores the distribution of pairwise distances among all sequences in the dataset looking for the gap between intra‐ and interspecific distances. To do so, distances are ranked and then a local slope function is computed given a window size to detect significant changes (i.e., increases) in the slope values that correspond to gaps in the initial distribution. Once the barcode gap is found, sequences are divided into groups (MOTUs) among which genetic distances are always larger than the gap distance that created the first local maximum slope. This is called the initial or primary partition. This process is then recursively applied to the groups found in the initial partition until no further splitting occurs. These new groups constitute the recursive partition. We used K2P and uncorrected distance matrices generated with MEGA as inputs and tested two relative gap width values (*X* = 1.0, 0.8). We registered the initial and recursive partitions for a range of prior intraspecific divergence (*P*) values between 0.001 (0.1%) and 0.1 (10%). Results were almost identical with the two distance metrics, but we observed a tendency to higher MOTU counts in the recursive partitions when *X* = 0.8. We, therefore, decided to take a more conservative approach and focus on the results obtained with K2P and *X* = 1.0.

TCS is commonly used to construct statistical parsimony haplotype networks. This method begins by estimating the maximum number of substitutions between two haplotypes as a result of single substitutions (i.e., avoiding homoplasy generated by multiple hits) under a certain probability of parsimony. Haplotypes are then connected to a network until the differences between them exceed the number of substitutions established by the parsimony limit. When the latter happens, the haplotypes end in different unconnected networks. The higher the cutoff value, the lower the number of substitutions allowed between haplotypes and the greater the count of unconnected networks generated. MOTU counts were recorded for ten different cutoff values (90%–99%) available in the software, but we focused on the MOTUs generated with the 95% cutoff value as this connection limit produced good results for real data in previous analyses (Hart & Sunday, 2007). Both for ABGD and TCS, all results can be found in S3 and S4.

Finally, RESL is the algorithm used to group COI barcode sequences uploaded to BOLD into genetic clusters (BINs) which constitute the Barcode Index Number system (Ratnasingham & Hebert, 2013). This method first divides the sequence alignment into initial MOTUs based on single linkage clustering with a threshold of 2.2% of maximum intracluster divergence. These primary MOTUs are then refined using Markov Clustering and the Silhouette Criterion (Ratnasingham & Hebert, 2013). BINs are generated using the information of the entire COI barcode library, so are not comparable with the MOTUs generated with ABGD and TCS. Therefore, we employed the RESL algorithm exclusively to our dataset using the Cluster Sequences analysis tool available on BOLD v4 (http://www.v4.boldsystems.org).

For the three methods described above, and to analyze the correspondence between reference species and MOTUs, each species was assigned to one of three categories: MATCH, SPLIT, or MERGE (Ratnasingham & Hebert, 2013). When all the specimens from a reference species were found to form a single MOTU, that species was placed in the MATCH category. When representatives of a species were divided into two or more MOTUs, it was assigned to the SPLIT category. Lastly, if members of two or more species were combined into a single MOTU, those species joined the MERGE category.

## 3 RESULTS

### 3.1 Ant diversity

We processed 623 specimens representing 182 species from 50 genera (Tables 1 and S1). Of these, 20% (37) represent new records for INP, and many of them constitute either a new record for Argentina (23) or a range expansion within the country (8) (Table S1). Among others, new records for the country include the arboreal termite specialist *Cylindromyrmex brasiliensis,* as well as *Megalomyrmex brandaoi, Neoponera curvinodis, Neoponera bactronica, Platythyrea pilosula, Procryptocerus adlerzi,* and *Leptogenys iheringi,* the latter collected carrying an isopod in its mandibles. There may be additional new taxa as we also recognized many morphospecies in genera for which the alpha taxonomy is not yet resolved (e.g., *Solenopsi*s, *Hypoponera, Pheidole, Neoponera*). Ant diversity currently includes 257 recognized species or morphospecies from 61 genera. An up‐to‐date checklist for the ant species of the INP is available on BOLD (CL‐INPA).

**Table 1:** The current number of species present at the Iguazú National Park (INP) and their representation in this study

Subfamily	Species at INP	Specimens/Species processed	Specimen/Species with sequences	Specimens/Species in the final dataset
Amblyoponinae	2	0	0	0
Dolichoderinae	16	32/11	19/8	18/8
Dorylinae	12	36/9	26/8	25/8
Ectatomminae	9	24/7	16/5	13/5
Formicinae	29	116/26	72/21	48/19
Heteroponerinae	4	11/3	7/3	7/3
Myrmicinae	136	283/88	139/56	129/52
Ponerinae	34	103/24	73/24	67/24
Proceratiinae	2	3/1	0	0
Pseudomyrmecinae	13	15/10	5/5	5/5
Total	257	623/182	357/130	312/124

### 3.2 Dataset and genetic distances

The final dataset used for the analyses consisted of 312 sequences from 124 species and 42 genera (Tables 1 and S2, https://doi.org/10.5883/ds-aoi16pub). On average, 2.5 sequences were analyzed per species (range 1–15), and the mean sequence length was 656 bp with 95% of the dataset corresponding to full barcode sequences (658 bp). We found two 3‐bp deletions in our alignment, one present in all individuals of *Dinoponera australis* starting at position 359, and the other in both *Apterostigma* morphospecies (PEH01 and PEH02) starting at position 473. Neither of these events altered the reading frame of the sequences. In fact, no stop codons were found, suggesting that no pseudogenes were amplified (Song, Buhay, Whiting, & Crandall, 2008). Similar cases have been reported for other hymenoptera (Hansson, Smith, Janzen, & Hallwachs, 2015; Quicke et al., 2012). In particular, comparing our results with other available sequences, this deletion is absent in *Dinoponera gigantea,* meanwhile, it appears to be present in all species of *Apterostigma*, with the exception of *A. megacephala* (Sosa‐Calvo et al., 2017).

**Figure 1:**
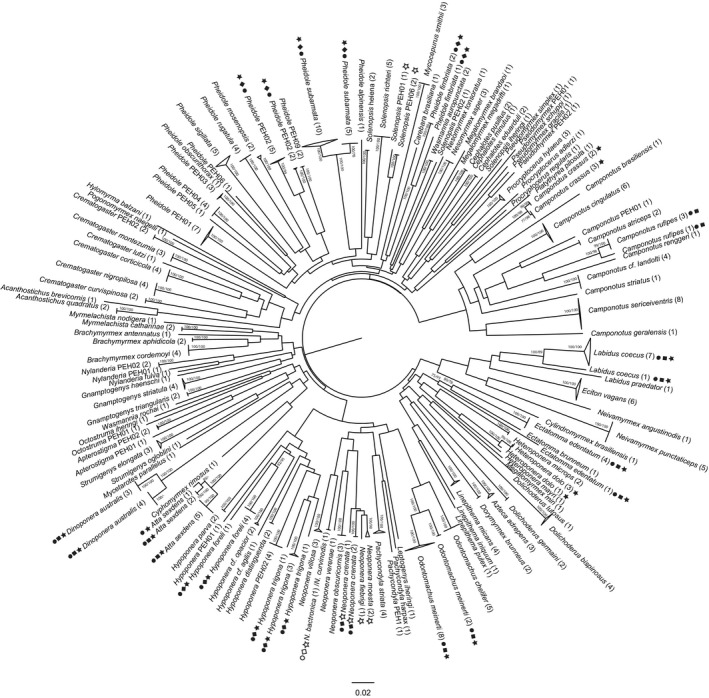
Neighbor‐joining (NJ) tree of 312 COI sequences of Iguazú National Park ants computed with a K2P substitution model (30 high‐quality sequences for specimens that were not identified to species were not included). Symbols next to the terminals indicate when a species was split (filled) or merged (blank) by RESL (circles), TCS (squares), or ABGD (stars). Numbers above the node correspond to NJ/ML (maximum likelihood) bootstrap support values based on 1,000 pseudoreplicates

**Figure 2:**
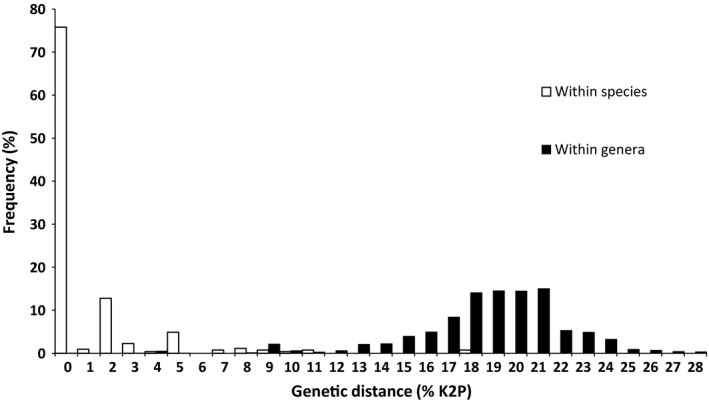
Frequency distribution of genetic distances within species and among congeneric species

**Figure 3:**
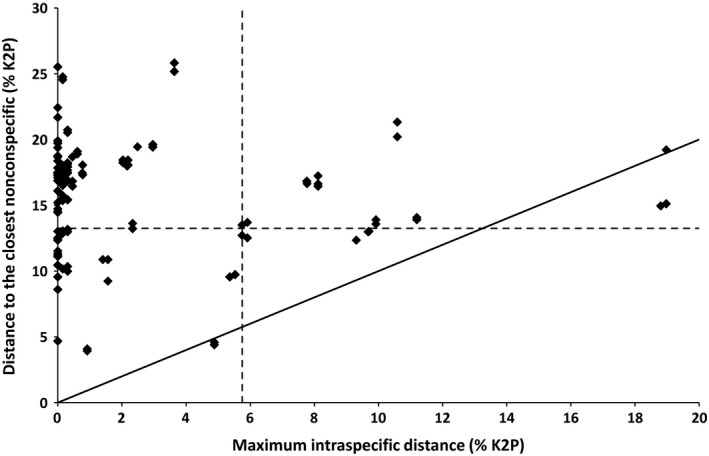
Barcode gap analysis for 65 species of ants with two or more individuals. Each individual is represented by a point, and the distance to the furthest heterospecific is plotted against the minimum distance to the nearest neighbor. The vertical dashed line shows the 95th percentile of all intraspecific distances (5.75%), while the horizontal one corresponds to the lower 5% of congeneric distances (13.25%). Points below the diagonal (1:1 relationship) correspond to *Ectatomma edentatum* and *Neoponera crenata* (see text for more details)

Based on 541 comparisons from 65 species (31 genera) with two or more individuals (253 sequences), the mean intraspecific distance was 0.72% (range 0.00%–7.57%; Figures 1 and 2). In contrast, and based on 2,502 comparisons among 283 pairs of congeneric species (110 species from 28 genera with two or more species), the mean congeneric distance was 17.25% (range 8.59%–25.22%; Figures 1 and 2), nearly 24 times larger than the mean intraspecific divergence. The average distance to the nearest neighbor (i.e., minimum interspecific distance) was 15.75% (range 0.00%–25.82%), almost eight times larger than 2.07%, the mean distance to the furthest intraspecific sequence (range 0.00%–18.97%). The lowest distance between two congeners was observed between *Neoponera bactronica* and *N. curvinodis,* which constitute the only case of barcode‐sharing (i.e., no sequence divergence) between species in our dataset. The second lowest interspecific distance (3.92%) was found between *Neoponera moesta* and *Neoponera fiebrigi*, while two specimens of *Ectatomma edentatum* showed the highest maximum intraspecific distance (18.97%). The distance to the furthest conspecific was always lower than the distance to the closest heterospecific for all species with more than one sequence, clearly showing the presence of a barcode gap. The only exceptions were *E. edentatum* and *Neoponera crenata* which fell below (or almost on) the 1:1 relationship line (Figure 3). These species were the only ones found to be paraphyletic according to the NJ tree (and the ML tree in the case of *N. crenata;* Figure 1). However, the paraphyly of *E. edentatum* was not recovered in the ML gene tree or a Bayesian topology (not shown).

### 3.3 Specimen identification simulations

The application of the BM criterion resulted in nearly 100% correct identifications with only one (0.40%) incorrect assignment (Table 2). The BCM criterion with a threshold of 5.75% (95th percentile of intraspecific distances) gave 97.23% of correct and 0.40% of incorrect identifications, and six queries (2.37%) remained unidentified (Table 2). The “threshVal” function suggested a threshold between 2.4% and 3.9% (Fig. S1), so we used the mean value (3.15%) for the analyses. With this threshold, the BCM approach delivered 97.23% of correct identifications, 2.77% (seven sequences) of unidentified queries, and no incorrect identifications (Table 2). The percentage of true identifications decreased slightly (96.05%, Table 2) when the threshold was set to lower divergences like the 1.26% suggested by the “localMinima” function in SPIDER (Fig. S2) and the BOLD's threshold (1%). In those cases, ten sequences (3.95%) did not have a match below the threshold (Table 2). The results with the BIC were identical to those obtained with the BCM criterion for the “threshVal,” “localMinima,” and BOLD's thresholds (Table 2). In the case of the 5.75% threshold, the BIC produced 94.86% of correct identifications, 2.77% ambiguous assignations, and six queries (2.37%) could not be identified (Table 2). Finally, it is worth mentioning that as singletons were excluded as queries, we did not include *N. bactronica* and *N. curvinodis*, the species pair that share their barcode sequence. If we had run these sequences against our database, we would have another two incorrect identifications under the BM and BCM criteria and two additional ambiguous assignments based on the BIC approach.

**Table 2:** Results of the sequence‐based identification simulations. Identifications were classified according to three criteria: Best Match (BM), Best Close Match (BCM), and BOLD Identification Criterion (BIC). For BCM and BIC approaches, we used four threshold values (5.75%, 3.15%, 1.26%, and 1.00%) obtained from different sources (see text). For the 253 queries that were run, we inform both the total number of identifications (within each category) and the percentage they represent (values in parenthesis)

Identification/Criterion	BM	95% percentile of intraspecific distances		“threshVal”		“localMinima”		BOLD's threshold	
		BCM (5.75%)	BIC (5.75%)	BCM (3.15%)	BIC (3.15%)	BCM (1.26%)	BIC (1.26%)	BCM (1.00%)	BIC (1.00%)
Correct	252 (99.60%)	246 (97.23%)	240 (94.86%)	246 (97.23%)	246 (97.23%)	243 (96.05%)	243 (96.05%)	243 (96.05%)	243 (96.05%)
Incorrect	1 (0.40%)	1 (0.40%)	–	–	–	–	–	–	–
Ambiguous	–	–	7 (2.77%)	–	–	–	–	–	–
No ID	–	6 (2.37%)	6 (2.37%)	7 (2.77%)	7 (2.77%)	10 (3.95%)	10 (3.95%)	10 (3.95%)	10 (3.95%)

When we queried the 30 sequences of males, minor workers, and queens that could not be identified to species based on morphology against both our database and the entire barcode library available on BOLD (as of January 2017), a species name was assigned to 22 (73%) of them based on BOLD's 1% threshold (Table 3). In each case, the closest match was a sequence that was part of this study's dataset. Three other cases showed a close match that also belonged to our database at divergence values between 2% and 3.8% (Table 3), casting doubt on whether the closest match was from the same species or not. For the remaining five sequences, the closest match was delivered by sequences from other projects available on BOLD, although genetic distances were between 6.1% and 14.15% (Table 3), suggesting that the species to which the unknown queries belong were not present in BOLD yet. In summary, 86% of the unknown sequences (25 of 30) had a close match provided by the records available in our project to barcode the ants of INP and 73% of those (22) resulted in species identification.

**Table 3:** Results of the sequence‐based specimen identification of 30 unidentified males, minor workers, and queens using the barcode database reported here and the entire barcode library available on BOLD. The table shows for each query the closest match, their sequence similarity, and the database in which that record was found. Matches with 99% or higher similarity constitute solid species identifications according to the BOLD Identification Criterion

Query			Closest match			
Process ID	Sample ID	Preliminary ID	Species ID	Process ID	Similarity (%)	Database
INSAR137‐11	MACN‐Bar‐Ins‐ct 00613	*Camponotus*	*Camponotus PEH01*	ANTPI403‐15	100.00	This study
INSAR716‐11	MACN‐Bar‐Ins‐ct 02539	*Camponotus*	*Camponotus PEH01*	ANTPI403‐15	100.00	This study
INSAR729‐11	MACN‐Bar‐Ins‐ct 02555	*Ectatomma*	*Ectatomma edentatum*	ANTPI017‐10	100.00	This study
INSAR746‐11	MACN‐Bar‐Ins‐ct 02573	*Ectatomma*	*Ectatomma edentatum*	ANTPI017‐10	100.00	This study
ANTPI185‐12	MACN‐Bar‐Ins‐ct 02968	*Camponotus*	*Camponotus rufipes*	ANTI106‐15	100.00	This study
ANTPI505‐15	MACN‐bar‐ins‐ct 06904	*Hypoponera*	*Hypoponera cf. opacior*	ANTPI249‐13	100.00	This study
ANTPI549‐15	MACN‐bar‐ins‐ct 06948	*Pheidole*	*Pheidole subarmata*	ANTPI009‐10	100.00	This study
INSAR493‐11	MACN‐Bar‐Ins‐ct 617	*Camponotus*	*Camponotus cingulatus*	ANTPI197‐13	100.00	This study
INSAR497‐11	MACN‐Bar‐Ins‐ct 621	*Camponotus*	*Camponotus cf. landolti*	ANTPI037‐10	100.00	This study
INSAR498‐11	MACN‐Bar‐Ins‐ct 622	*Camponotus*	*Camponotus cf. landolti*	ANTPI037‐10	100.00	This study
INSAR499‐11	MACN‐Bar‐Ins‐ct 623	*Camponotus*	*Camponotus cf. landolti*	ANTPI037‐10	100.00	This study
INSAR500‐11	MACN‐Bar‐Ins‐ct 624	*Camponotus*	*Camponotus cf. landolti*	ANTPI037‐10	100.00	This study
INSAR501‐11	MACN‐Bar‐Ins‐ct 625	*Camponotus*	*Camponotus cingulatus*	ANTPI197‐13	100.00	This study
INSAR508‐11	MACN‐Bar‐Ins‐ct 632	*Camponotus*	*Camponotus cf. landolti*	ANTPI037‐10	100.00	This study
INSAR510‐11	MACN‐Bar‐Ins‐ct 635	*Camponotus*	*Camponotus cf. landolti*	ANTPI037‐10	100.00	This study
INSAR511‐11	MACN‐Bar‐Ins‐ct 636	*Camponotus*	*Camponotus cingulatus*	ANTPI197‐13	100.00	This study
ANTPI479‐15	MACN‐bar‐ins‐ct 06878	*Hypoponera*	*Hypoponera trigona*	ANTI133‐15	99.85	This study
INSAR492‐11	MACN‐Bar‐Ins‐ct 616	*Camponotus*	*Camponotus cingulatus*	ANTPI197‐13	99.84	This study
INSAR494‐11	MACN‐Bar‐Ins‐ct 618	*Camponotus*	*Camponotus cingulatus*	ANTPI197‐13	99.84	This study
INSAR507‐11	MACN‐Bar‐Ins‐ct 631	*Camponotus*	*Camponotus cingulatus*	ANTPI197‐13	99.69	This study
INSAR495‐11	MACN‐Bar‐Ins‐ct 619	*Camponotus*	*Camponotus cingulatus*	ANTI166‐15	99.53	This study
INSAR738‐11	MACN‐Bar‐Ins‐ct 02564	*Neoponera*	*Neoponera crenata*	ANTPI410‐15	99.08	This study
INSAR509‐11	MACN‐Bar‐Ins‐ct 634	*Camponotus*	*Camponotus PEH01*	ANTPI403‐15	97.98	This study
INSAR745‐11	MACN‐Bar‐Ins‐ct 02572	*Neoponera*	*Neoponera crenata*	ANTI101‐15	96.64	This study
ANTI173‐15	MACN‐bar‐ins‐ct 06470	*Neivamyrmex*	*Neivamyrmex angustinodis*	ANTPI409‐15	96.18	This study
INSAR751‐11	MACN‐Bar‐Ins‐ct 02580	*Dorymyrmex*	*Dorymyrmex sp. CIP01*	NA	93.88	BOLD
INSAR491‐11	MACN‐Bar‐Ins‐ct 614	*Camponotus*	*Camponotus EC07*	DRYLO063‐15	90.28	BOLD
INSAR512‐11	MACN‐Bar‐Ins‐ct 637	*Camponotus*	*Camponotus EC07*	DRYLO063‐15	90.28	BOLD
INSAR752‐11	MACN‐Bar‐Ins‐ct 02581	Formicinae	*Brachymyrmex cordemoyi*	NA	88.12	BOLD
ANTPI558‐15	MACN‐bar‐ins‐ct 06957	Ectatomminae	*Gnamptogenys annulata*	NA	85.85	BOLD

### 3.4 MOTUs delineation analyses

**Figure 4:**
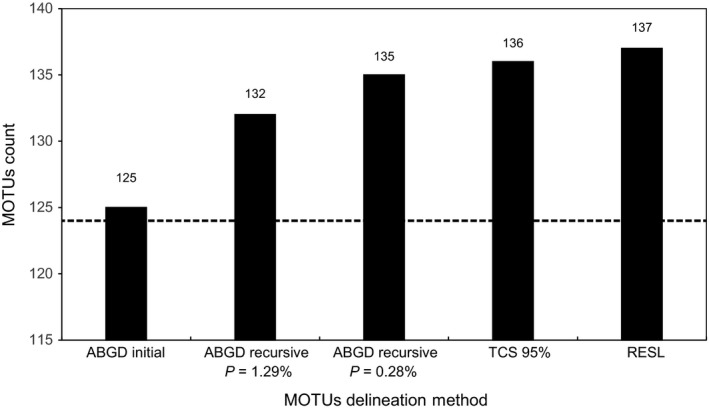
Number of MOTUs obtained for each clustering delimitation methodology. Dashed line represents the number of identified species (124).

The MOTU counts obtained with the three clustering methods and the setting parameters ranged from 125 to 137 (Table S2, Figure 4). Therefore, all methods delivered MOTU counts higher than the number of reference species (124; Table S2, Figure 4).

**Figure 5:**
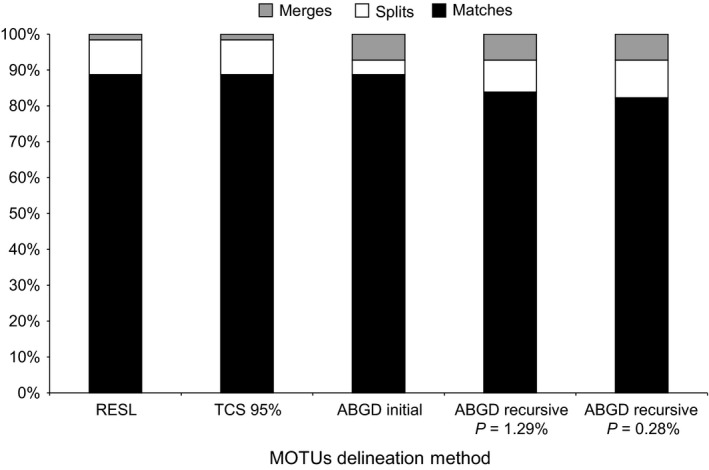
Percentages of MATCHES, SPLITS AND MERGES for the different clustering methods discussed in the text based on the correspondence between reference species and MOTUs boundaries.

The RESL algorithm found 137 MOTUs, a 10% increase on the number of reference species in our dataset (Figure 4). In terms of MOTU composition, 89% were MATCHES and 10% were SPLITS, while two species (1.61%) were merged into a single MOTU (Table S2; Figure 5). The latter corresponds to *N. bactronica* and *N. curvinodis*, the barcode‐sharing species pair that was always merged into one MOTU regardless of the method (Table 4). Additionally, twelve species (10%) were divided into two or more genetic clusters by RESL (Table 4, Figure 1): *Atta sexdens* and *Hypoponera trigona* were the only two species split into three MOTUs, while the remaining 10 species were divided into two (Table 4). All of these species showed elevated intraspecific divergences with mean distances always above 1% and average maximum intraspecific distances over 2% (Table 4). Six of these species (Table 4) showed distances to the furthest conspecific that were higher (or equal) than the 95th percentile of intraspecific distances (5.75%).

**Table 4:** Summary of 22 species split or merged by at least one of the methodologies used. Sampling size (*N*), mean and maximum intraspecific distance, and minimum distance to the nearest neighbor are indicated. MATCH, SPLIT, and MERGE categories indicate the correspondence between the boundaries of the species and that of the MOTUs delineated by RESL, TCS, and ABGD (numbers in parenthesis indicate the MOTU count). In the case of ABGD, we inform the results obtained with the initial partition and two recursive partitions chosen based on different criteria (see text for more details). Numbers in brackets after the SPLIT category indicate the number of groups in which the species was divided. A complete table with all the species analyzed can be found in Table S2

Species (22)	*N*	Mean distance (% K2P)	Max distance (% K2P)	Min distance to NN (% K2P)	RESL (137)	TCS 95% (136)	ABGD initial partition (125)	ABGD recursive *P* = 1.29% (132)	ABGD recursive *P* = 0.28% (135)
*Atta sexdens*	8	1.60	2.97	19.42	SPLIT (3)	SPLIT (2)	MATCH	SPLIT (3)	SPLIT (3)
*Camponotus crassus*	5	0.46	0.78	17.31	MATCH	MATCH	MATCH	MATCH	SPLIT (2)
*Camponotus rufipes*	4	1.17	2.33	13.22	SPLIT (2)	SPLIT (2)	MATCH	MATCH	MATCH
*Dinoponera australis*	7	2.08	3.64	25.16	SPLIT (2)	SPLIT (2)	MATCH	SPLIT (2)	SPLIT (2)
*Ectatomma edentatum*	5	7.57	18.97	14.95	SPLIT (2)	SPLIT (2)	SPLIT (2)	SPLIT (2)	SPLIT (2)
*Heteroponera dolo*	4	0.81	1.57	9.23	MATCH	MATCH	MATCH	SPLIT (2)	SPLIT (2)
*Hypoponera foreli*	5	4.48	11.20	13.90	SPLIT (2)	SPLIT (2)	SPLIT (2)	SPLIT (2)	SPLIT (2)
*Hypoponera trigona*	5	6.41	9.92	12.35	SPLIT (3)	SPLIT (3)	SPLIT (3)	SPLIT (3)	SPLIT (3)
*Labidus coecus*	8	2.32	8.12	16.44	SPLIT (2)	SPLIT (2)	SPLIT (2)	SPLIT (2)	SPLIT (4)
*Neoponera bactronica*	1	NA	NA	0.00	MERGE	MERGE	MERGE	MERGE	MERGE
*Neoponera crenata*	3	3.26	4.88	4.39	SPLIT (2)	SPLIT (2)	MERGE	MERGE	MERGE
*Neoponera curvinodis*	1	NA	NA	0.00	MERGE	MERGE	MERGE	MERGE	MERGE
*Neoponera fiebrigi*	1	NA	NA	3.92	MATCH	MATCH	MERGE	MERGE	MERGE
*Neoponera moesta*	2	0.92	0.92	3.92	MATCH	MATCH	MERGE	MERGE	MERGE
*Odontomachus meinerti*	10	1.96	5.53	9.55	SPLIT (2)	SPLIT (2)	MATCH	SPLIT (2)	SPLIT (2)
*Pheidole fimbriata*	3	7.06	10.59	20.19	SPLIT (2)	SPLIT (2)	SPLIT (2)	SPLIT (2)	SPLIT (2)
*Pheidole PEH02*	7	2.75	5.91	12.52	SPLIT (2)	SPLIT (2)	MATCH	SPLIT (2)	SPLIT (2)
*Pheidole subarmata*	15	1.09	2.19	17.99	SPLIT (2)	SPLIT (2)	MATCH	SPLIT (2)	SPLIT (2)
*Pseudomyrmex gracilis*	1	NA	NA	5.84	MATCH	MATCH	MERGE	MERGE	MERGE
*Pseudomyrmex PEH02*	1	NA	NA	5.84	MATCH	MATCH	MERGE	MERGE	MERGE
*Solenopsis PEH01*	1	NA	NA	4.67	MATCH	MATCH	MERGE	MERGE	MERGE
*Solenopsis PEH06*	2	0.00	0.00	4.67	MATCH	MATCH	MERGE	MERGE	MERGE

The 136 MOTUs delineated by TCS with the 95% cutoff value represents an increase of 10% in the number of reference species (Figure 4; Table S3). The percentages of MATCHES, MERGES, and SPLITS were identical to those of RESL (Table S2, Figure 5) and the same twelve species were also split into two genetic clusters, being the only difference was that *Atta sexdens* was split into two MOTUs with TCS, instead of three (Table 4, Figure 1).

Automatic Barcode Gap Discovery produced a single initial partition that consisted of 125 MOTUs (Table S4, Figure 4), the count closest to the number of reference species (124). When we inspected the MOTU composition of this initial partition, we observed almost the same percentage (88.71%) of MATCHES as in RESL and TCS, but a higher proportion of MERGES (7.26%) and a lower incidence (4.03%) of SPLITS (Table S2, Figure 5). Five of the twelve species divided by TCS and RESL were also split in ABGD's initial partition (Table 4, Figure 1). In terms of recursive partitions, extremely low *p* values (.1%) produced MOTU counts strikingly higher than the number of reference species due to oversplitting, while extremely high values (10%) lumped all species into a single group (Table S4). It is worth noting that ABGD was not only the algorithm with the highest incidence of MERGES, but also the only method to merge two or more reference species (other than the barcode‐sharing species of *Neoponera*) into a single cluster (Table 4).

**Figure 6:**
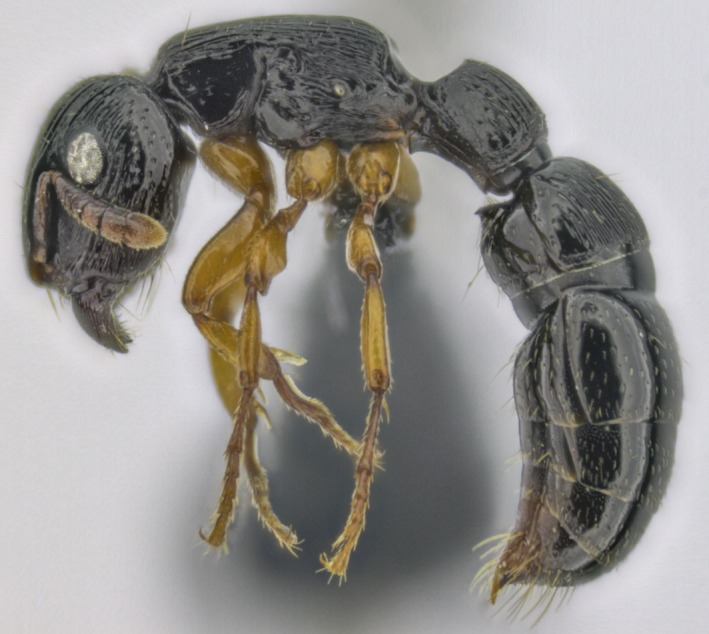
One of the new records of Iguazú National Park: the arboreal termite hunter, *Cylindromyrmex brasiliensis*

Puillandre et al. (2012) found that a prior value around 1% showed the greatest correspondence between the number of MOTUs and that of reference species for different datasets. In our study, a prior value of 1.29% produced a recursive partitioning scheme that consisted of 132 MOTUs (a 6% increase compared to the number of reference species; Table S4, Figure 4). This MOTU count was also the median number of clusters generated across all prior values (Table S4), and this prior is very similar to the threshold used for specimen identification by BOLD (1%) and almost identical to that suggested by the “localMinima” function (1.26%), both of which resulted in a high percentage of correct identifications. However, these MOTUs showed a lower correspondence between their boundaries and those of the reference species with 84% of MATCHES, 9% SPLITS, and 7% MERGES (Table 4, Figure 5). In addition, the prior values between 0.17% and 0.46% delivered 135 MOTUs, the closest count to those of the other methods (Table 4). These MOTUs represent an increase of 9% compared to the number of reference species (Figure 4) and included 83% of MATCHES, 10% of SPLITS, and 7% of MERGES (Table S2, Figure 5). These two recursive partitioning schemes split the same twelve species that TCS and RESL, with the exception of *Camponotus rufipes* and *N. crenata* (Table 4, Figure 1). Additionally, with *p* = .17%–.46% (Table 4, Figure 1), *Labidus coecus* and *Camponotus crassus* were divided into four and two groups, respectively. Lastly, *Heteroponera dolo* was recovered as two distinct MOTUs in both recursive partitioning schemes (Table 4, Figure 1).

To conclude, it is worth mentioning that the MOTUs that were identified within species by the three methodologies employed (i.e., intraspecific splits) showed in most cases high bootstrap support, ranging from 73% to 100% and being over 95% in 80% of the cases (Figure 1).

## 4 DISCUSSION

### 4.1 Dataset and genetic distances

We assembled a DNA barcode reference library consisting of 312 COI sequences for 124 species of ants from the southernmost region of the Atlantic Forest. This dataset covers nearly 50% of the ant species known for INP. Despite that over 600 specimens were collected, only 50% of them made it to the final dataset. Our low amplification success may be associated with old samples (up to 10 years, Table S1) and sample storage conditions that were not DNA‐friendly (e.g., ants collected with pitfall traps and litter samples). If these problematic samples are not considered, the amplification success increases to 74%.

Mean interspecific divergence was markedly higher than that registered within species in the ants of INP. More importantly, all species but two (*E. edentatum* and *N. crenata*) had higher distances to their closest heterospecific than to the furthest conspecific, providing evidence of a clear barcode gap for almost all the species represented by at least two sequences. A comparable study of the Ants of Coco Island (but with fewer specimens) showed intra and interspecific mean divergence values similar to those reported here (0.58% and 27%, respectively; Smith, Hallwachs, Janzen, & Segura, 2013). It should be noted, however, that our values for intraspecific variation may be underestimated due to the relatively low number of sequences per species. For instance, the mean intraspecific divergence among species represented by at least five individuals was 1.74%, being markedly higher than that among species with two to four specimens (0.33%). Congruently, we found a positive relationship (*p *<* *.05) between sampling size and mean intraspecific divergence, although the association was weak (Pearson correlation: *R* ^2^=0.07, *r* = .26). However, many of the species that evidenced high intraspecific distances may represent cryptic species (Table 4 and see Section 3.2 below), so they might not be true representatives of intraspecific variation. Future studies should focus on assessing the real extent of cryptic diversity to achieve a better comprehension of species boundaries before obtaining a new estimate of intraspecific variation for the ants of INP.

### 4.2 Specimen identification

We simulated a sequenced‐based identification process to test the utility of our DNA barcode library with three different identification criteria. Most (from 94% to 99%) of the ant species surveyed in this study (represented by at least two sequences) can be identified regardless of the criteria or threshold used (Table 3). Singletons were also distinguishable as they all possessed unique (i.e., not shared) DNA barcodes that allowed their discrimination from the closest heterospecific in the gene trees, with the exception of *N. bactronica* and *N. curvinodis* which constitute the only case of barcode‐sharing between species in our dataset. In fact, these two species were recorded for the first time in Argentina and are members of a species complex that is difficult to identify (Fernandes et al., 2014; Lucas et al., 2002). They can be separated mainly by the anterior petiolar face (curved in *N. curvinodis*) (Fernandes et al., 2014). Possible variation of this character might difficult the identification of these specimens at its southern distribution.

For species assignments, we explored the use of four different sequence divergence thresholds (5.75%, 3.15%, 1.26%, and 1%). Nonetheless, hardly a single distance threshold can be universally applied. For example, a range of 0%–14% of divergence in COI has been found for the ant species *Crematogaster kelleri* (Blaimer, Fisher, Feldhaar, Mackay, & Nei, 2013) in Madagascar. The identification success decreased only slightly when using lower thresholds (1%–1.26%) compared to higher thresholds (3.15%–5.75%). This reflects the fact that higher thresholds can aid in the identification of species with high intraspecific variation. However, as stated before, species with deep intraspecific divergence could represent two or more cryptic taxa. Taking this into consideration, the lower thresholds (1%–1.26%) could be suitable for ant species discrimination, the identification of intraspecific lineages, and the generation of cryptic species hypotheses, an important step in the process of the discovery and description of diversity (Seifert, 2009).

Our study was focused on a particular area of the Atlantic Forest, but it would be worth evaluating if a lower threshold compromises the identification success of the ants of Argentina (or Southern South America) as the geographic coverage increases. For example a study of 1,000 species of European Lepidoptera found that large geographic distances had a small impact on genetic intraspecific variation and therefore, on the performance of DNA Barcodes (Huemer et al., 2014). The advantage of using higher versus lower thresholds will depend on various factors, including the level of intraspecific variation (that in turn depends on the organisms studied and possibly the extent of geographic coverage) and the presence of cryptic species in the group analyzed. This is why we consider that using a range of thresholds and comparing their results as we did here is the best option to assess diversity and also further understand the characteristics of the organisms under study.

We were unable to identify, based on external morphology alone, 30 specimens (mostly males and queens) captured in light traps. As these specimens were successfully sequenced, we used them to assess whether the database assembled here and the complete DNA barcode library available on BOLD could assign a species name to them. Twenty‐five queries (86%) had a close match that was part of our barcode library, and 73% of them resulted in species identification (less than 1% of divergence between the query and the match). None of the identified males have been formally described, illustrating the difficulty in identifying species based on males alone. This also highlights the usefulness of DNA barcode libraries in general as an identification tool and in particular for linking reproductive castes with workers, facilitating their subsequent description, and inclusion in taxonomic keys (e.g., Yoshimura & Fisher, 2007). The fact that all the species name assignments came from our own database reflect the current underrepresentation of ants of the Atlantic Forest in BOLD and emphasize the need for increasing the geographic coverage of the global library in order to fully benefit from the use of DNA barcodes.

### 4.3 MOTU delineation

In terms of composition, all methodologies delivered a high percentage of matches, close to 90% (Figure 5). ABGD's initial partition resulted in the MOTU count closest to the number of reference species. This is not surprising as primary partitions are typically stable on a wider range of prior values and are normally close to the number of taxonomic species (Puillandre et al., 2012). At the same time, ABGD was the only algorithm to lump into the same MOTU species that form clearly distinct clades in the NJ and ML trees (Table 4, Figures 1 and 5). This method merged *Neoponera moesta* and *N. fiebrigi* with *N. crenata*, and P*seudomyrmex gracilis* with *Pseudomyrmex PEH02*, and the two morphospecies *Solenopsis PEH01* and *Solenopsis PEH06* (Table 4, Figure 1). This may be a consequence of the small number of samples for these species; Puillandre et al. (2012) suggested that ABGD works better when there are more than 3–5 sequences per species. In our dataset, almost 50% of the sequences correspond to singletons (59) and, five of eight species involved in cases of MERGE are represented by only one sequence (Table 4, Figure 1). As for TCS and RESL, it is not clear if a high presence of singletons might affect the performance of the clustering algorithms, although, Ratnasingham and Hebert (2013) showed that the performance of RESL does not vary greatly across datasets with varying sampling densities. All three methods split 10 species into two or more MOTUs (Table 4, Figure 1), while *Camponotus rufipes* was split by RESL and TCS but not ABGD, and *Heteroponera dolo* and *Camponotus crassus* were split into two MOTUs only by ABGD.

### 4.4 Cases of high intraspecific variation and ant diversity

Among species with high intraspecific variation in our dataset, six species had distances higher than the 95th percentile of intraspecific distances (5.75%): *E. edentatum, Hypoponera foreli, P. fimbriata, H. trigona, L. coecus,* and *Pheidole* PEH02 with a maximum intraspecific divergence of 18.97%, 11.20%, 10.59%, 9.92%, 8.12%, and 5.91%, respectively. *Ectatomma edentatum* was split into two MOTUs with one cluster being composed of four individuals with an identical barcode sequence and the other one consisting of only one specimen (MACN‐Bar‐Ins‐ct06433). Additionally, this species was paraphyletic in the NJ tree (Figure 1). These two MOTUs can be distinguished morphologically by the interruption of the striate sculpture around the spiracle of the third abdominal segment and the petiole shape (Fig. S3); the type material of *E. edentatum* appears to correspond with the morphotype of MACN‐Bar‐Ins‐ct06433 (ANTWEB https://www.antweb.org/), with a taller petiole depressed from the sides (lateral view). This pattern persists even when additional DNA barcoded specimens are included from other localities in Misiones province (Hanisch, unpublished), suggesting that these MOTUs indeed represent different species that are not currently recognized with available taxonomic keys. We also found that *H. foreli, P. fimbriata, L. coecus*,* H. trigona,* and *Pheidole* PEH02 were also split into two or more MOTUs, but we were unable to find any external morphological traits that support these intraspecific genetic clusters.

Among species with moderate intraspecific variation, three ponerine species standout: *Odontomachus meinerti, N. crenata,* and *D. australis*, with a maximum intraspecific divergence of 5.53%, 4.48%, and 3.64%, respectively. Additionally, *N. crenata* was one of the two cases where the barcode gap was absent (Figure 3). These values may reflect the variation in reproductive and dispersal strategies (Peeters & Ito, 2001). For example, *Dinoponera* lacks a winged queen caste, which is usually associated with low dispersion and subsequently more marked genetic structure. Moreover, an unknown male identified using our library as *N. crenata* (MACN‐Bar‐Ins‐ct 02564; Table 3) lacked the characteristic subpetiolar process of the species (Fig. S4; Mackay & Mackay, 2010), suggesting that both the unknown male and the matching *N. crenata* could actually be other species that currently keys out as *N. crenata*.

The incidence of cryptic species might be high in ants (Seifert, 2009), and evidence of cryptic species has been reported recently for genera included in this study. For example, Aguilar‐Velasco et al. (2016) using morphology and both nuclear and mitochondrial loci found that *Ectatomma ruidum* is a complex of at least three different species. Similarly, Barth, Moritz, and Kraus (2015) used morphological and genetic characters to identify cryptic species in Mexican populations of *Labidus praedator*. In our study, deep intraspecific divergence is currently supported in most cases only by COI data. Therefore, alternative explanations need to be considered. For example, high sequence divergence among morphologically similar specimens could arise as a consequence of infection with the maternally transmitted endosymbiont *Wolbachia* (Smith et al., 2012), although its prevalence has been shown to be generally low (Smith et al., 2012). In a similar manner, the co‐amplification of pseudogenes could lead to false conclusions (Song et al., 2008), especially in those cases where one of the intraspecific divergent lineages is represented by a single individual. We examined our sequences in search for characteristics that might indicate the presence of pseudogenes, including insertions or deletions that altered the reading frame, biased base compositions, excess of nonsynonymous substitutions, and the presence of stop codons. Even though our assessment showed no evidence of the co‐amplification of pseudogenes, further studies should look into these possibilities in more detail as more specimens become available, given that sometimes pseudogenes can be cryptic and lack insertions, deletions, or frame shift mutations (Kerr, 2010).

In January 2017, our records (including the 30 unidentified specimens) were assigned to 144 BINs on BOLD, being 78 of them new to the database. This represents a significant addition to the DNA barcode reference library of the ants of South America. At the same time, our sampling resulted in 37 additions to the species list of INP, with 23 of them representing first records for Argentina. The number of MOTUs estimated with three different clustering algorithms was always higher than the number of species identified based on morphology, suggesting the existence of cryptic diversity. If these cases do reflect new species, the diversity of ants at the INP could be between 6% and 10% higher than currently recognized. Moreover, because nearly half of our sequences are represented by singletons, the extent of cryptic diversity may be underestimated. In conclusion, our study supports the use of clustering algorithms to explore biodiversity and that DNA barcodes can be useful for ant species identification and caste association. We encourage further studies to integrate genetic evidence with morphological data in order to get a better understanding of ant diversity in southern South America in general and the Atlantic Forest in particular.

## CONFLICT OF INTEREST

None declared.

## DATA ACCESSIBILITY

All collection and sequence data are available in BOLD in the public datasets “DS‐AOI16ALL” (https://doi.org/10.5883/ds-aoi16all), “DS‐AOI16PUB” (https://doi.org/10.5883/ds-aoi16pub) and in GenBank (accession numbers MF925738–MF926049). Iguazú National Park checklist can be found at BOLD (CL‐INPA).

## AUTHOR CONTRIBUTIONS

PEH collected, identified, and processed the samples, performed data analysis, and wrote the manuscript. PDL performed the genetic analyses and wrote the manuscript. ML provided samples and revised the manuscript. CIP and AVS provided samples and revised the manuscript. DAL and PLT managed the project and revised the manuscript. PEH, PDL, and CIP conceived and designed the study.
