# Supplementary material for: Mind the gap! Integrating taxonomic approaches to assess ant diversity at the southern extreme of the Atlantic Forest
Source: Ecol Evol. 2017 Nov 10;7(23):10451–66. doi: 10.1002/ece3.3549 (PMC5723617; doi:10.1002/ece3.3549)
Supplement: Supplementary file 1 [file ECE3-7-10451-s001.docx]

**Supplementary material**

Table S1. Summary of 623 specimens processed for this study. For each individual, we provide the Sample and Process IDs along with the year of collection. For those individuals that were successfully sequenced we provide information on the length of the COI sequence together with the GenBank accession numbers and BIN information. (A) new record for INP checklist (*) new record for Misiones province, (**) new record for Argentina

| Identification | Sample ID | Process ID | GenBank | BIN | Sequence length (bp) | Collection year |
| --- | --- | --- | --- | --- | --- | --- |
| *Acanthostichus brevicornis* | T6S6a2009PNIG02 | ANTPI074-10 | MF925975 | BOLD:ABU8923 | 600[0n] | 2009 |
| *Acanthostichus brevicornis* | T2S16a2008PNIC11 | ANTPI035-10 |  |  | 0 | 2008 |
| *Acanthostichus brevicornis* | MACN-Bar-Ins-ct 04968 | ANTPI268-13 |  |  | 0 | 2009 |
| *Acanthostichus brevicornis* | T6S6m2009PNIF05 | ANTPI065-10 |  |  | 0 | 2009 |
| *Acanthostichus quadratus* | MACN-bar-ins-ct 06431 | ANTI134-15 | MF925767 | BOLD:ACZ3360 | 658[0n] | 2015 |
| *Acanthostichus quadratus* | MACN-bar-ins-ct 06922 | ANTPI523-15 | MF926028 | BOLD:ACZ3360 | 658[0n] | 2015 |
| *Acromyrmex hispidus* | MACN-bar-ins-ct 06961 | ANTPI562-15 |  |  | 0 | 1998 |
| *Acromyrmex laticeps* | MACN-Bar-Ins-ct 04979 | ANTPI193-13 |  |  | 0 | 2011 |
| *Acromyrmex subterraneus (A)* | MACN-Bar-Ins-ct 06796 | ANTPI397-15 |  |  | 0 | 1998 |
| *Acromyrmex subterraneus (A)* | MACN-Bar-Ins-ct 06832 | ANTPI433-15 |  |  | 0 | 1999 |
| *Acromyrmex subterraneus (A)* | MACN-Bar-Ins-ct 06842 | ANTPI443-15 |  |  | 0 | 1999 |
| *Acromyrmex subterraneus (A)* | MACN-bar-ins-ct 06478 | ANTI181-15 |  |  | 0 | 2015 |
| *Apterostigma PEH01* | MACN-bar-ins-ct 06479 | ANTI182-15 | MF925927 | BOLD:ACZ3911 | 603[0n] | 2015 |
| *Apterostigma PEH02* | MACN-bar-ins-ct 06468 | ANTI171-15 | MF925864 | BOLD:ACZ4237 | 658[0n] | 2015 |
| *Apterostigma PEH02* | MACN-bar-ins-ct 06482 | ANTI185-15 | MF925785 | BOLD:ACZ4237 | 658[0n] | 2015 |
| *Apterostigma PEH02* | MACN-Bar-Ins-ct 06782 | ANTPI383-15 |  |  | 0 | 1999 |
| *Apterostigma PEH02* | MACN-Bar-Ins-ct 06805 | ANTPI406-15 |  |  | 0 | 1999 |
| *Apterostigma PEH02* | MACN-Bar-Ins-ct 06836 | ANTPI437-15 |  |  | 0 | 1999 |
| *Apterostigma PEH02* | MACN-Bar-Ins-ct 06846 | ANTPI447-15 |  |  | 0 | 1999 |
| *Atta sexdens* | MACN-Bar-Ins-ct 06814 | ANTPI415-15 | MF925972 | BOLD:ABV3852 | 658[0n] | 1998 |
| *Atta sexdens* | MACN-Bar-Ins-ct 06860 | ANTPI461-15 | MF925770 | BOLD:ABV3852 | 658[0n] | 1998 |
| *Atta sexdens* | MACN-Bar-Ins-ct 02514 | INSAR655-11 | MF925924 | BOLD:ABV3852 | 658[0n] | 2011 |
| *Atta sexdens* | MACN-Bar-Ins-ct 02515 | INSAR656-11 | MF925939 | BOLD:ABV3852 | 658[0n] | 2011 |
| *Atta sexdens* | MACN-Bar-Ins-ct 02517 | INSAR658-11 | MF925806 | BOLD:ABV3852 | 658[0n] | 2011 |
| *Atta sexdens* | MACN-Bar-Ins-ct 02912 | ANTPI129-12 | MF925926 | BOLD:ACC4094 | 658[0n] | 2011 |
| *Atta sexdens* | MACN-Bar-Ins-ct 04974 | ANTPI191-13 | MF925819 | BOLD:ACC4094 | 658[0n] | 2011 |
| *Atta sexdens* | MACN-bar-ins-ct 06443 | ANTI146-15 | MF925797 | BOLD:ACZ4391 | 658[0n] | 2015 |
| *Atta sexdens* | MACN-Bar-Ins-ct 02516 | INSAR657-11 |  |  | 658[0n] | 2011 |
| *Atta sexdens* | MACN-bar-ins-ct 06958 | ANTPI559-15 |  |  | 0 | 2015 |
| *Azteca adrepens* | MACN-Bar-Ins-ct 04976 | ANTPI192-13 | MF925946 | BOLD:ACM2309 | 658[0n] | 2011 |
| *Azteca adrepens* | MACN-Bar-Ins-ct 04982 | ANTPI194-13 | MF925771 | BOLD:ACM2309 | 658[0n] | 2011 |
| *Azteca adrepens* | MACN-bar-ins-ct 06918 | ANTPI519-15 | MF925892 | BOLD:ACM2309 | 658[0n] | 2011 |
| *Azteca adrepens* | MACN-Bar-Ins-ct 04980 | ANTPI271-13 |  |  | 0 | 2011 |
| *Azteca alfari* | MACN-Bar-Ins-ct 02915 | ANTPI132-12 |  |  | 0 | 2009 |
| *Basiceros disciger* | MACN-bar-ins-ct 06959 | ANTPI560-15 |  |  | 658[0n] | 1998 |
| *Brachymyrmex antennatus* | T4S10a2009PNIE04 | ANTPI052-10 | MF925990 | BOLD:AAU4116 | 658[0n] | 2009 |
| *Brachymyrmex antennatus* | MACN-Bar-Ins-ct 04984 | ANTPI196-13 |  |  | 0 | 2009 |
| *Brachymyrmex aphidicola* | T3S12A2009PNIG10 | ANTPI082-10 | MF925827 | BOLD:AAU4115 | 658[0n] | 2009 |
| *Brachymyrmex aphidicola* | T3S12m2009PNIC08 | ANTPI032-10 | MF925929 | BOLD:AAU4115 | 658[0n] | 2009 |
| *Brachymyrmex aphidicola* | MACN-Bar-Ins-ct 06801 | ANTPI402-15 |  |  | 0 | 1999 |
| *Brachymyrmex aphidicola* | T3P42008PNIH09 | ANTPI093-10 |  |  | 0 | 2008 |
| *Brachymyrmex aphidicola* | T3W52008PNID06 | ANTPI042-10 |  |  | 0 | 2008 |
| *Brachymyrmex aphidicola* | T6S4a2009PNID09 | ANTPI045-10 |  |  | 0 | 2009 |
| *Brachymyrmex cordemoyi* | MACN-Bar-Ins-ct 02950 | ANTPI167-12 | MF925761 | BOLD:ACC4473 | 658[0n] | 2011 |
| *Brachymyrmex cordemoyi* | MACN-Bar-Ins-ct 02961 | ANTPI178-12 | MF925900 | BOLD:ACC4473 | 658[0n] | 2011 |
| *Brachymyrmex cordemoyi* | MACN-Bar-Ins-ct 02970 | ANTPI187-12 | MF925855 | BOLD:ACC4473 | 658[0n] | 2011 |
| *Brachymyrmex cordemoyi* | MACN-Bar-Ins-ct 04983 | ANTPI195-13 | MF925989 | BOLD:ACC4473 | 658[0n] | 2011 |
| *Brachymyrmex cordemoyi* | MACN-bar-ins-ct 06912 | ANTPI513-15 |  |  | 0 | 2005 |
| *Brachymyrmex cordemoyi* | MACN-bar-ins-ct 06936 | ANTPI537-15 |  |  | 0 | 2005 |
| *Brachymyrmex cordemoyi* | MACN-bar-ins-ct 06952 | ANTPI553-15 |  |  | 0 | 2005 |
| *Brachymyrmex PEH01* | MACN-Bar-Ins-ct 06781 | ANTPI382-15 |  |  | 0 | 1999 |
| *Brachymyrmex PEH01* | MACN-Bar-Ins-ct 06822 | ANTPI423-15 |  |  | 0 | 1999 |
| *Brachymyrmex PEH01* | MACN-Bar-Ins-ct 06863 | ANTPI464-15 |  |  | 0 | 1999 |
| *Camponotus* | MACN-Bar-Ins-ct 634 | INSAR509-11 |  | BOLD:AAI3890 | 658[0n] | 2010 |
| *Camponotus* | MACN-Bar-Ins-ct 02968 | ANTPI185-12 |  | BOLD:AAI3891 | 658[0n] | 2011 |
| *Camponotus* | MACN-Bar-Ins-ct 621 | INSAR497-11 |  | BOLD:AAW7847 | 658[0n] | 2010 |
| *Camponotus* | MACN-Bar-Ins-ct 622 | INSAR498-11 |  | BOLD:AAW7847 | 658[0n] | 2010 |
| *Camponotus* | MACN-Bar-Ins-ct 623 | INSAR499-11 |  | BOLD:AAW7847 | 658[0n] | 2010 |
| *Camponotus* | MACN-Bar-Ins-ct 624 | INSAR500-11 |  | BOLD:AAW7847 | 658[0n] | 2010 |
| *Camponotus* | MACN-Bar-Ins-ct 632 | INSAR508-11 |  | BOLD:AAW7847 | 658[0n] | 2010 |
| *Camponotus* | MACN-Bar-Ins-ct 635 | INSAR510-11 |  | BOLD:AAW7847 | 658[0n] | 2010 |
| *Camponotus* | MACN-Bar-Ins-ct 614 | INSAR491-11 |  | BOLD:AAZ4158 | 658[0n] | 2010 |
| *Camponotus* | MACN-Bar-Ins-ct 637 | INSAR512-11 |  | BOLD:AAZ4158 | 658[0n] | 2010 |
| *Camponotus* | MACN-Bar-Ins-ct 616 | INSAR492-11 |  | BOLD:AAZ4159 | 658[0n] | 2010 |
| *Camponotus* | MACN-Bar-Ins-ct 617 | INSAR493-11 |  | BOLD:AAZ4159 | 658[0n] | 2010 |
| *Camponotus* | MACN-Bar-Ins-ct 618 | INSAR494-11 |  | BOLD:AAZ4159 | 658[0n] | 2010 |
| *Camponotus* | MACN-Bar-Ins-ct 619 | INSAR495-11 |  | BOLD:AAZ4159 | 658[0n] | 2010 |
| *Camponotus* | MACN-Bar-Ins-ct 625 | INSAR501-11 |  | BOLD:AAZ4159 | 658[0n] | 2010 |
| *Camponotus* | MACN-Bar-Ins-ct 631 | INSAR507-11 |  | BOLD:AAZ4159 | 658[0n] | 2010 |
| *Camponotus* | MACN-Bar-Ins-ct 636 | INSAR511-11 |  | BOLD:AAZ4159 | 658[0n] | 2010 |
| *Camponotus* | MACN-Bar-Ins-ct 00613 | INSAR137-11 |  | BOLD:ABY9693 | 658[0n] | 2010 |
| *Camponotus* | MACN-Bar-Ins-ct 02539 | INSAR716-11 |  | BOLD:ABY9693 | 658[0n] | 2011 |
| *Camponotus* | MACN-Bar-Ins-ct 00633 | INSAR139-11 |  |  | 403[0n] | 2010 |
| *Camponotus* | MACN-Bar-Ins-ct 05001 | ANTPI203-13 |  |  | 0 | 2011 |
| *Camponotus* | MACN-Bar-Ins-ct 05733 | INSAR1397-15 |  |  | 0 | 2014 |
| *Camponotus atriceps* | MACN-Bar-Ins-ct 628 | INSAR504-11 | MF925795 | BOLD:AAW0798 | 658[0n] | 2010 |
| *Camponotus atriceps* | MACN-Bar-Ins-ct 629 | INSAR505-11 | MF925921 | BOLD:AAW0798 | 658[0n] | 2010 |
| *Camponotus atriceps* | MACN-Bar-Ins-ct 02910 | ANTPI127-12 |  |  | 0 | 2008 |
| *Camponotus atriceps* | T6P52008PNIE05 | ANTPI053-10 |  |  | 0 | 2008 |
| *Camponotus brasiliensis (**)* | MACN-bar-ins-ct 06454 | ANTI157-15 | MF925780 | BOLD:ACZ3824 | 658[0n] | 2015 |
| *Camponotus brasiliensis (**)* | MACN-bar-ins-ct 06903 | ANTPI504-15 |  |  | 0 | 2015 |
| *Camponotus cf. landolti* | T6P12008PNID07 | ANTPI043-10 | MF926048 | BOLD:AAW7847 | 658[0n] | 2008 |
| *Camponotus cf. landolti* | T6P22008PNID01 | ANTPI037-10 | MF925805 | BOLD:AAW7847 | 658[0n] | 2008 |
| *Camponotus cf. landolti* | MACN-Bar-Ins-ct 02892 | ANTPI109-12 | MF926044 | BOLD:AAW7847 | 658[0n] | 2011 |
| *Camponotus cf. landolti* | MACN-Bar-Ins-ct 02906 | ANTPI123-12 | MF925749 | BOLD:AAW7847 | 658[0n] | 2011 |
| *Camponotus cf. landolti* | MACN-Bar-Ins-ct 04999 | ANTPI201-13 |  |  | 0 | 2008 |
| *Camponotus cf. landolti* | T1P12008PNIF01 | ANTPI061-10 |  |  | 0 | 2008 |
| *Camponotus cf. landolti* | T1P32008PNIF07 | ANTPI067-10 |  |  | 0 | 2008 |
| *Camponotus cf. landolti* | T1P52008PNIG11 | ANTPI083-10 |  |  | 0 | 2008 |
| *Camponotus cingulatus* | MACN-Bar-Ins-ct 02927 | ANTPI144-12 | MF926037 | BOLD:AAZ4159 | 658[0n] | 2011 |
| *Camponotus cingulatus* | MACN-Bar-Ins-ct 04994 | ANTPI197-13 | MF925763 | BOLD:AAZ4159 | 658[0n] | 2011 |
| *Camponotus cingulatus* | MACN-Bar-Ins-ct 04996 | ANTPI198-13 | MF925838 | BOLD:AAZ4159 | 658[0n] | 2011 |
| *Camponotus cingulatus* | MACN-Bar-Ins-ct 04998 | ANTPI200-13 | MF925815 | BOLD:AAZ4159 | 658[0n] | 2011 |
| *Camponotus cingulatus* | MACN-bar-ins-ct 06429 | ANTI132-15 | MF925877 | BOLD:AAZ4159 | 658[0n] | 2015 |
| *Camponotus cingulatus* | MACN-bar-ins-ct 06463 | ANTI166-15 | MF925987 | BOLD:AAZ4159 | 658[0n] | 2015 |
| *Camponotus cingulatus* | MACN-Bar-Ins-ct 04992 | ANTPI273-13 |  |  | 0 | 2008 |
| *Camponotus cingulatus* | T2P12008PNIE07 | ANTPI055-10 |  |  | 0 | 2008 |
| *Camponotus cingulatus* | T3P42008PNIG03 | ANTPI075-10 |  |  | 0 | 2008 |
| *Camponotus crassus* | MACN-Bar-Ins-ct 02880 | ANTPI097-12 | MF925960 | BOLD:AAW1347 | 658[0n] | 2011 |
| *Camponotus crassus* | MACN-Bar-Ins-ct 02894 | ANTPI111-12 | MF925917 | BOLD:AAW1347 | 658[0n] | 2011 |
| *Camponotus crassus* | MACN-Bar-Ins-ct 02920 | ANTPI137-12 | MF925944 | BOLD:AAW1347 | 658[0n] | 2011 |
| *Camponotus crassus* | MACN-Bar-Ins-ct 02951 | ANTPI168-12 | MF925745 | BOLD:AAW1347 | 650[0n] | 2011 |
| *Camponotus crassus* | MACN-Bar-Ins-ct 04997 | ANTPI199-13 | MF925933 | BOLD:AAW1347 | 658[0n] | 2011 |
| *Camponotus crassus* | MACN-Bar-Ins-ct 02904 | ANTPI121-12 |  |  | 658[0n] | 2011 |
| *Camponotus depressus* | MACN-Bar-Ins-ct 05144 | ANTPI253-13 |  |  | 0 | 2011 |
| *Camponotus depressus* | MACN-bar-ins-ct 06437 | ANTI140-15 |  |  | 0 | 2015 |
| *Camponotus geralensis (A)* | MACN-Bar-Ins-ct 626 | INSAR502-11 | MF925851 | BOLD:AAZ4160 | 658[0n] | 2010 |
| *Camponotus lespessi* | T2P32008PNIC10 | ANTPI034-10 |  |  | 285[0n] | 2008 |
| *Camponotus lespessi* | MACN-Bar-Ins-ct 02886 | ANTPI103-12 |  |  | 0 | 2011 |
| *Camponotus lespessi* | MACN-Bar-Ins-ct 02922 | ANTPI139-12 |  |  | 0 | 2011 |
| *Camponotus PEH01* | MACN-Bar-Ins-ct 06802 | ANTPI403-15 | MF925993 | BOLD:ABY9693 | 658[0n] | 1999 |
| *Camponotus PEH01* | MACN-Bar-Ins-ct 06807 | ANTPI408-15 |  |  | 0 | 1999 |
| *Camponotus punctulatus* | MACN-bar-ins-ct 06960 | ANTPI561-15 |  |  | 0 | 1998 |
| *Camponotus renggeri* | MACN-Bar-Ins-ct 02948 | ANTPI165-12 | MF925982 | BOLD:ACC4407 | 658[0n] | 2011 |
| *Camponotus renggeri* | T4P22008PNID04 | ANTPI040-10 |  |  | 0 | 2008 |
| *Camponotus rufipes* | MACN-bar-ins-ct 06403 | ANTI106-15 | MF925750 | BOLD:AAI3891 | 658[0n] | 2015 |
| *Camponotus rufipes* | MACN-Bar-Ins-ct 02884 | ANTPI101-12 | MF925843 | BOLD:ACC4111 | 658[0n] | 2011 |
| *Camponotus rufipes* | MACN-Bar-Ins-ct 02898 | ANTPI115-12 | MF925831 | BOLD:ACC4111 | 658[0n] | 2011 |
| *Camponotus rufipes* | MACN-Bar-Ins-ct 02964 | ANTPI181-12 | MF925739 | BOLD:ACC4111 | 658[0n] | 2011 |
| *Camponotus rufipes* | MACN-Bar-Ins-ct 06798 | ANTPI399-15 |  |  | 0 | 1999 |
| *Camponotus scissus* | MACN-bar-ins-ct 06964 | ANTPI565-15 |  |  | 0 | 1998 |
| *Camponotus sericeiventris* | T2P42008PNIF04 | ANTPI064-10 | MF925756 | BOLD:AAW7849 | 658[0n] | 2008 |
| *Camponotus sericeiventris* | MACN-Bar-Ins-ct 02518 | INSAR659-11 | MF925768 | BOLD:AAW7849 | 658[0n] | 2011 |
| *Camponotus sericeiventris* | MACN-Bar-Ins-ct 02882 | ANTPI099-12 | MF925868 | BOLD:AAW7849 | 658[0n] | 2011 |
| *Camponotus sericeiventris* | MACN-Bar-Ins-ct 02908 | ANTPI125-12 | MF925812 | BOLD:AAW7849 | 658[0n] | 2011 |
| *Camponotus sericeiventris* | MACN-Bar-Ins-ct 02916 | ANTPI133-12 | MF925898 | BOLD:AAW7849 | 658[0n] | 2011 |
| *Camponotus sericeiventris* | MACN-Bar-Ins-ct 02929 | ANTPI146-12 | MF925862 | BOLD:AAW7849 | 658[0n] | 2011 |
| *Camponotus sericeiventris* | MACN-Bar-Ins-ct 02940 | ANTPI157-12 | MF925800 | BOLD:AAW7849 | 658[0n] | 2011 |
| *Camponotus sericeiventris* | MACN-Bar-Ins-ct 02972 | ANTPI189-12 | MF925897 | BOLD:AAW7849 | 658[0n] | 2011 |
| *Camponotus sericeiventris* | MACN-Bar-Ins-ct 05731 | INSAR1395-15 |  |  | 0 | 2014 |
| *Camponotus striatus (**)* | T6P22008PNIE11 | ANTPI059-10 | MF926007 | BOLD:AAW1349 | 658[2n] | 2008 |
| *Camponotus trapezoideus* | MACN-bar-ins-ct 06882 | ANTPI483-15 |  |  | 0 | 2015 |
| *Carebara brasiliana* | MACN-bar-ins-ct 06937 | ANTPI538-15 | MF925889 | BOLD:ACZ3340 | 658[0n] | 2003 |
| *Carebara brasiliana* | MACN-bar-ins-ct 06949 | ANTPI550-15 |  |  | 0 | 2003 |
| *Carebara brevipilosa* | MACN-Bar-Ins-ct 06790 | ANTPI391-15 |  |  | 0 | 1999 |
| *Carebara brevipilosa* | MACN-Bar-Ins-ct 06813 | ANTPI414-15 |  |  | 0 | 1999 |
| *Carebara brevipilosa* | MACN-Bar-Ins-ct 06831 | ANTPI432-15 |  |  | 0 | 1999 |
| *Carebara brevipilosa* | MACN-Bar-Ins-ct 06852 | ANTPI453-15 |  |  | 0 | 1999 |
| *Carebara brevipilosa* | MACN-Bar-Ins-ct 05000 | ANTPI202-13 |  |  | 0 | 2009 |
| *Carebara brevipilosa* | T5S4a2009PNIF12 | ANTPI072-10 |  |  | 0 | 2009 |
| *Carebara brevipilosa* | T5S4m2009PNIG04 | ANTPI076-10 |  |  | 485[8n] | 2009 |
| *Cephalotes eduarduli (A)* | MACN-bar-ins-ct 06422 | ANTI125-15 | MF926016 | BOLD:ACQ0707 | 658[0n] | 2015 |
| *Cephalotes eduarduli (A)* | MACN-bar-ins-ct 06967 | ANTPI568-16 | MF926034 | BOLD:ACQ0707 | 658[0n] | 2015 |
| *Cephalotes minutus* | MACN-bar-ins-ct 06455 | ANTI158-15 | MF925879 | BOLD:ACY4404 | 658[0n] | 2015 |
| *Cephalotes pusillus* | MACN-Bar-Ins-ct 05151 | ANTPI259-13 | MF925985 | BOLD:ACM2165 | 658[0n] | 2011 |
| *Cephalotes pusillus* | MACN-Bar-Ins-ct 05153 | ANTPI261-13 | MF925867 | BOLD:ACM2165 | 658[0n] | 2011 |
| *Cephalotes pusillus* | MACN-Bar-Ins-ct 05004 | ANTPI275-13 |  |  | 0 | 2008 |
| *Crematogaster cl. obscurata (**)* | MACN-bar-ins-ct 06954 | ANTPI555-15 |  |  | 0 | 2015 |
| *Crematogaster corticicola* | MACN-bar-ins-ct 06449 | ANTI152-15 | MF925802 | BOLD:ACN2394 | 658[0n] | 2015 |
| *Crematogaster corticicola* | MACN-bar-ins-ct 06887 | ANTPI488-15 | MF925980 | BOLD:ACN2394 | 658[0n] | 2015 |
| *Crematogaster corticicola* | MACN-bar-ins-ct 07019 | ANTPI620-16 | MF926013 | BOLD:ACN2394 | 658[0n] | 2016 |
| *Crematogaster corticicola* | MACN-bar-ins-ct 07057 | ANTPI658-16 | MF925836 | BOLD:ACN2394 | 658[0n] | 2016 |
| *Crematogaster crinosa* | MACN-bar-ins-ct 06442 | ANTI145-15 |  |  | 0 | 2015 |
| *Crematogaster curvispinosa (*)* | MACN-bar-ins-ct 06461 | ANTI164-15 | MF925901 | BOLD:ACZ3040 | 658[0n] | 2015 |
| *Crematogaster curvispinosa (*)* | MACN-bar-ins-ct 06945 | ANTPI546-15 | MF925943 | BOLD:ACZ3040 | 566[0n] | 2015 |
| *Crematogaster erecta* | MACN-Bar-Ins-ct 05157 | ANTPI265-13 |  |  | 0 | 2008 |
| *Crematogaster lutzi (**)* | MACN-Bar-Ins-ct 06838 | ANTPI439-15 | MF925978 | BOLD:ACZ3057 | 545[3n] | 1998 |
| *Crematogaster lutzi (**)* | MACN-Bar-Ins-ct 06803 | ANTPI404-15 |  |  | 0 | 1998 |
| *Crematogaster montezumia* | MACN-bar-ins-ct 06427 | ANTI130-15 | MF925904 | BOLD:ACZ3938 | 658[0n] | 2015 |
| *Crematogaster montezumia* | MACN-bar-ins-ct 07013 | ANTPI614-16 | MF925858 | BOLD:ACZ3938 | 658[0n] | 2015 |
| *Crematogaster montezumia* | MACN-bar-ins-ct 07042 | ANTPI643-16 | MF925783 | BOLD:ACZ3938 | 658[0n] | 2015 |
| *Crematogaster nigropilosa* | MACN-Bar-Ins-ct 02890 | ANTPI107-12 | MF925922 | BOLD:ACC4305 | 658[0n] | 2011 |
| *Crematogaster nigropilosa* | MACN-Bar-Ins-ct 02896 | ANTPI113-12 | MF925905 | BOLD:ACC4305 | 658[0n] | 2011 |
| *Crematogaster nigropilosa* | MACN-Bar-Ins-ct 02942 | ANTPI159-12 | MF925748 | BOLD:ACC4305 | 658[0n] | 2011 |
| *Crematogaster nigropilosa* | MACN-Bar-Ins-ct 02953 | ANTPI170-12 | MF925813 | BOLD:ACC4305 | 658[0n] | 2011 |
| *Crematogaster nigropilosa* | MACN-Bar-Ins-ct 06784 | ANTPI385-15 |  |  | 0 | 1999 |
| *Crematogaster nigropilosa* | T4P12008PNIH01 | ANTPI085-10 |  |  | 0 | 2008 |
| *Crematogaster nigropilosa* | MACN-Bar-Ins-ct 02924 | ANTPI141-12 |  |  | 658[0n] | 2011 |
| *Crematogaster PEH01* | MACN-Bar-Ins-ct 06795 | ANTPI396-15 |  |  | 0 | 1999 |
| *Crematogaster PEH01* | MACN-Bar-Ins-ct 06830 | ANTPI431-15 |  |  | 0 | 1999 |
| *Crematogaster PEH01* | MACN-Bar-Ins-ct 06848 | ANTPI449-15 |  |  | 0 | 1999 |
| *Crematogaster PEH01* | MACN-Bar-Ins-ct 02888 | ANTPI105-12 |  |  | 0 | 2011 |
| *Crematogaster PEH01* | MACN-bar-ins-ct 06475 | ANTI178-15 |  |  | 0 | 2011 |
| *Crematogaster PEH01* | MACN-bar-ins-ct 06965 | ANTPI566-15 |  |  | 0 | 2011 |
| *Crematogaster PEH02* | MACN-Bar-Ins-ct 627 | INSAR503-11 | MF926025 | BOLD:AAZ4161 | 658[0n] | 2010 |
| *Crematogaster PEH02* | MACN-Bar-Ins-ct 630 | INSAR506-11 | MF926040 | BOLD:AAZ4161 | 658[0n] | 2010 |
| *Cylindromyrmex brasiliensis (**)* | MACN-bar-ins-ct 07036 | ANTPI637-16 | MF925940 | BOLD:ADE2377 | 658[0n] | 2015 |
| *Cyphomyrmex minutus* | MACN-Bar-Ins-ct 06788 | ANTPI389-15 |  |  | 0 | 1999 |
| *Cyphomyrmex minutus* | MACN-Bar-Ins-ct 06792 | ANTPI393-15 |  |  | 0 | 1999 |
| *Cyphomyrmex minutus* | MACN-Bar-Ins-ct 06815 | ANTPI416-15 |  |  | 0 | 1999 |
| *Cyphomyrmex minutus* | MACN-Bar-Ins-ct 06826 | ANTPI427-15 |  |  | 0 | 1999 |
| *Cyphomyrmex minutus* | MACN-Bar-Ins-ct 06833 | ANTPI434-15 |  |  | 0 | 1999 |
| *Cyphomyrmex minutus* | MACN-bar-ins-ct 06939 | ANTPI540-15 |  |  | 0 | 2015 |
| *Cyphomyrmex olitor* | MACN-Bar-Ins-ct 06820 | ANTPI421-15 |  |  | 0 | 1999 |
| *Cyphomyrmex rimosus* | MACN-bar-ins-ct 06420 | ANTI123-15 | MF925959 | BOLD:ACM5106 | 658[0n] | 2015 |
| *Dinoponera australis* | MACN-Bar-Ins-ct 00615 | INSAR138-11 | MF925772 | BOLD:AAV4568 | 658[0n] | 2010 |
| *Dinoponera australis* | MACN-Bar-Ins-ct 02519 | INSAR660-11 | MF926022 | BOLD:AAV4568 | 658[0n] | 2011 |
| *Dinoponera australis* | MACN-Bar-Ins-ct 02520 | INSAR661-11 | MF925799 | BOLD:AAV4568 | 658[0n] | 2011 |
| *Dinoponera australis* | MACN-Bar-Ins-ct 02521 | INSAR662-11 | MF925847 | BOLD:AAV4568 | 658[0n] | 2011 |
| *Dinoponera australis* | MACN-Bar-Ins-ct 02973 | ANTPI190-12 | MF925936 | BOLD:ACC4124 | 658[0n] | 2011 |
| *Dinoponera australis* | MACN-bar-ins-ct 06394 | ANTI097-15 | MF925893 | BOLD:ACC4124 | 658[0n] | 2015 |
| *Dinoponera australis* | MACN-bar-ins-ct 06405 | ANTI108-15 | MF926009 | BOLD:ACC4124 | 658[0n] | 2015 |
| *Dinoponera australis* | T6P22008PNID05 | ANTPI041-10 |  |  | 0 | 2008 |
| *Dinoponera australis* | MACN-Bar-Ins-ct 05752 | INSAR1416-15 |  |  | 0 | 2012 |
| *Discothyrea sexarticulata (**)* | MACN-Bar-Ins-ct 06804 | ANTPI405-15 |  |  | 0 | 1999 |
| *Discothyrea sexarticulata (**)* | MACN-Bar-Ins-ct 06865 | ANTPI466-15 |  |  | 0 | 1999 |
| *Discothyrea sexarticulata (**)* | MACN-Bar-Ins-ct 06872 | ANTPI473-15 |  |  | 0 | 1999 |
| *Dolichoderus bispinosus* | MACN-Bar-Ins-ct 02959 | ANTPI176-12 | MF925995 | BOLD:ACC4406 | 658[0n] | 2008 |
| *Dolichoderus bispinosus* | MACN-Bar-Ins-ct 05010 | ANTPI270-13 | MF925853 | BOLD:ACC4406 | 658[0n] | 2008 |
| *Dolichoderus bispinosus* | MACN-Bar-Ins-ct 05008 | ANTPI204-13 | MF925886 | BOLD:ACC4406 | 658[0n] | 2011 |
| *Dolichoderus bispinosus* | MACN-Bar-Ins-ct 05152 | ANTPI260-13 | MF925791 | BOLD:ACC4406 | 658[0n] | 2011 |
| *Dolichoderus germaini (**)* | MACN-bar-ins-ct 06408 | ANTI111-15 | MF926049 | BOLD:ACZ4117 | 658[0n] | 2015 |
| *Dolichoderus germaini (**)* | MACN-bar-ins-ct 06451 | ANTI154-15 | MF925890 | BOLD:ACZ4117 | 658[0n] | 2015 |
| *Dolichoderus lutosus (**)* | MACN-bar-ins-ct 06894 | ANTPI495-15 | MF925909 | BOLD:ACZ2733 | 658[0n] | 2015 |
| *Dorymyrmex* | MACN-Bar-Ins-ct 02580 | INSAR751-11 |  | BOLD:ABV2662 | 658[0n] | 2011 |
| *Dorymyrmex brunneus* | MACN-Bar-Ins-ct 02933 | ANTPI150-12 | MF925937 | BOLD:ACC4210 | 658[0n] | 2008 |
| *Dorymyrmex brunneus* | MACN-Bar-Ins-ct 02955 | ANTPI172-12 | MF925849 | BOLD:ACC4210 | 658[0n] | 2008 |
| *Eciton vagans* | MACN-Bar-Ins-ct 600 | INSAR486-11 | MF925824 | BOLD:AAX9809 | 658[0n] | 2010 |
| *Eciton vagans* | MACN-Bar-Ins-ct 602 | INSAR488-11 | MF925793 | BOLD:AAX9809 | 658[0n] | 2010 |
| *Eciton vagans* | MACN-Bar-Ins-ct 603 | INSAR489-11 | MF925920 | BOLD:AAX9809 | 658[0n] | 2010 |
| *Eciton vagans* | MACN-Bar-Ins-ct 604 | INSAR490-11 | MF925790 | BOLD:AAX9809 | 658[0n] | 2010 |
| *Eciton vagans* | MACN-bar-ins-ct 06452 | ANTI155-15 | MF925962 | BOLD:AAX9809 | 658[0n] | 2015 |
| *Eciton vagans* | MACN-bar-ins-ct 06879 | ANTPI480-15 | MF926003 | BOLD:AAX9809 | 649[0n] | 2015 |
| *Eciton vagans* | MACN-Bar-Ins-ct 601 | INSAR487-11 |  |  | 0 | 2010 |
| *Ectatomma* | MACN-Bar-Ins-ct 02555 | INSAR729-11 |  | BOLD:AAX9859 | 658[0n] | 2011 |
| *Ectatomma* | MACN-Bar-Ins-ct 02573 | INSAR746-11 |  | BOLD:AAX9859 | 658[0n] | 2011 |
| *Ectatomma brunneum* | MACN-bar-ins-ct 06446 | ANTI149-15 | MF925777 | BOLD:ACX3889 | 658[0n] | 2015 |
| *Ectatomma edentatum* | T2W42008PNIB05 | ANTPI017-10 | MF925846 | BOLD:AAX9859 | 658[0n] | 2008 |
| *Ectatomma edentatum* | MACN-Bar-Ins-ct 02949 | ANTPI166-12 | MF925782 | BOLD:AAX9859 | 658[0n] | 2011 |
| *Ectatomma edentatum* | MACN-Bar-Ins-ct 05011 | ANTPI205-13 | MF925969 | BOLD:AAX9859 | 658[1n] | 2011 |
| *Ectatomma edentatum* | MACN-Bar-Ins-ct 05123 | ANTPI243-13 | MF925829 | BOLD:AAX9859 | 658[0n] | 2011 |
| *Ectatomma edentatum* | MACN-bar-ins-ct 06433 | ANTI136-15 | MF925817 | BOLD:ACZ3750 | 658[0n] | 2015 |
| *Ectatomma edentatum* | MACN-Bar-Ins-ct 02931 | ANTPI148-12 |  |  | 0 | 2008 |
| *Ectatomma edentatum* | T3P52008PNIH06 | ANTPI090-10 |  |  | 0 | 2008 |
| Ectatomminae | MACN-bar-ins-ct 06957 | ANTPI558-15 |  | BOLD:ABV2806 | 658[0n] | 2015 |
| Formicinae | MACN-Bar-Ins-ct 02581 | INSAR752-11 |  | BOLD:ABV2661 | 658[0n] | 2011 |
| *Gnamptogenys haenschi* | MACN-bar-ins-ct 06413 | ANTI116-15 | MF925963 | BOLD:ACZ4223 | 658[0n] | 2015 |
| *Gnamptogenys hartmani (*)* | MACN-bar-ins-ct 06924 | ANTPI525-15 |  |  | 0 | 2015 |
| *Gnamptogenys PEH01* | MACN-bar-ins-ct 06415 | ANTI118-15 |  |  | 0 | 2015 |
| *Gnamptogenys PEH01* | MACN-bar-ins-ct 06448 | ANTI151-15 |  |  | 0 | 2015 |
| *Gnamptogenys striatula* | MACN-Bar-Ins-ct 02944 | ANTPI161-12 | MF926038 | BOLD:ACM2117 | 658[0n] | 2011 |
| *Gnamptogenys striatula* | MACN-Bar-Ins-ct 05014 | ANTPI206-13 | MF925896 | BOLD:ACM2117 | 658[0n] | 2011 |
| *Gnamptogenys striatula* | MACN-Bar-Ins-ct 05124 | ANTPI244-13 | MF925914 | BOLD:ACM2117 | 658[0n] | 2011 |
| *Gnamptogenys striatula* | MACN-Bar-Ins-ct 05149 | ANTPI257-13 | MF925758 | BOLD:ACM2117 | 658[0n] | 2011 |
| *Gnamptogenys striatula* | MACN-Bar-Ins-ct 05016 | ANTPI277-13 |  |  | 0 | 2008 |
| *Gnamptogenys striatula* | T5P32008PNIH10 | ANTPI094-10 |  |  | 0 | 2008 |
| *Gnamptogenys striatula* | MACN-Bar-Ins-ct 02914 | ANTPI131-12 |  |  | 0 | 2011 |
| *Gnamptogenys triangularis (*)* | MACN-bar-ins-ct 06425 | ANTI128-15 | MF925778 | BOLD:ACZ3490 | 658[0n] | 2015 |
| *Gnamptogenys triangularis (*)* | MACN-bar-ins-ct 06900 | ANTPI501-15 | MF926026 | BOLD:ACZ3490 | 658[0n] | 2015 |
| *Heteroponera dolo* | MACN-Bar-Ins-ct 02946 | ANTPI163-12 | MF926014 | BOLD:ACC4116 | 658[0n] | 2011 |
| *Heteroponera dolo* | MACN-Bar-Ins-ct 05020 | ANTPI208-13 | MF925951 | BOLD:ACC4116 | 658[0n] | 2011 |
| *Heteroponera dolo* | MACN-bar-ins-ct 06438 | ANTI141-15 | MF925792 | BOLD:ACC4116 | 649[1n] | 2015 |
| *Heteroponera dolo* | MACN-bar-ins-ct 06472 | ANTI175-15 | MF926008 | BOLD:ACC4116 | 658[0n] | 2015 |
| *Heteroponera dolo* | MACN-Bar-Ins-ct 05017 | ANTPI207-13 |  |  | 0 | 2011 |
| *Heteroponera mayri* | MACN-Bar-Ins-ct 06824 | ANTPI425-15 | MF926021 | BOLD:ACZ3308 | 658[0n] | 1998 |
| *Heteroponera mayri* | MACN-Bar-Ins-ct 06857 | ANTPI458-15 |  |  | 0 | 1998 |
| *Heteroponera mayri* | MACN-Bar-Ins-ct 06867 | ANTPI468-15 |  |  | 0 | 1998 |
| *Heteroponera mayri* | MACN-Bar-Ins-ct 06812 | ANTPI413-15 |  |  | 0 | 1999 |
| *Heteroponera microps* | T5S10m2009PNIE12 | ANTPI060-10 | MF925787 | BOLD:AAV7129 | 658[0n] | 2009 |
| *Heteroponera microps* | T6S6a2009PNIF08 | ANTPI068-10 | MF925965 | BOLD:AAV7129 | 658[0n] | 2009 |
| *Hylomyrma balzani* | MACN-bar-ins-ct 06926 | ANTPI527-15 | MF925822 | BOLD:ACZ3727 | 658[0n] | 2015 |
| *Hylomyrma balzani* | MACN-Bar-Ins-ct 06791 | ANTPI392-15 |  |  | 0 | 1999 |
| *Hylomyrma balzani* | MACN-Bar-Ins-ct 05095 | ANTPI274-13 |  |  | 0 | 2008 |
| *Hylomyrma PEH01* | MACN-Bar-Ins-ct 06841 | ANTPI442-15 |  |  | 0 | 1999 |
| *Hylomyrma reitteri* | MACN-Bar-Ins-ct 06868 | ANTPI469-15 |  |  | 0 | 1998 |
| *Hylomyrma reitteri* | MACN-Bar-Ins-ct 06799 | ANTPI400-15 |  |  | 0 | 1999 |
| *Hylomyrma reitteri* | MACN-Bar-Ins-ct 06818 | ANTPI419-15 |  |  | 0 | 1999 |
| *Hypoponera* | MACN-bar-ins-ct 06878 | ANTPI479-15 |  | BOLD:AAU1873 | 658[0n] | 2005 |
| *Hypoponera* | MACN-bar-ins-ct 06904 | ANTPI505-15 |  | BOLD:ACM2976 | 658[0n] | 2003 |
| *Hypoponera cf. agilis (**)* | MACN-bar-ins-ct 06897 | ANTPI498-15 | MF926020 | BOLD:ACZ2699 | 651[0n] | 2005 |
| *Hypoponera cf. agilis (**)* | MACN-bar-ins-ct 06929 | ANTPI530-15 |  |  | 0 | 2005 |
| *Hypoponera cf. opacior* | MACN-bar-ins-ct 06895 | ANTPI496-15 | MF926018 | BOLD:ACM2976 | 658[0n] | 2008 |
| *Hypoponera cf. opacior* | MACN-Bar-Ins-ct 05140 | ANTPI249-13 | MF925934 | BOLD:ACM2976 | 658[0n] | 2011 |
| *Hypoponera cf. opacior* | MACN-bar-ins-ct 06888 | ANTPI489-15 |  |  | 0 | 2003 |
| *Hypoponera cf. opacior* | MACN-bar-ins-ct 06901 | ANTPI502-15 |  |  | 0 | 2005 |
| *Hypoponera distinguenda* | MACN-Bar-Ins-ct 05026 | ANTPI210-13 | MF925885 | BOLD:ACM2219 | 658[0n] | 2011 |
| *Hypoponera distinguenda* | MACN-bar-ins-ct 06441 | ANTI144-15 | MF926019 | BOLD:ACM2219 | 658[0n] | 2015 |
| *Hypoponera distinguenda* | MACN-Bar-Ins-ct 06844 | ANTPI445-15 |  |  | 0 | 1999 |
| *Hypoponera distinguenda* | T1W32008PNID12 | ANTPI048-10 |  |  | 0 | 2008 |
| *Hypoponera distinguenda* | MACN-Bar-Ins-ct 05028 | ANTPI280-13 |  |  | 0 | 2015 |
| *Hypoponera foreli* | T6W32008PNIF06 | ANTPI066-10 | MF925970 | BOLD:AAU1875 | 658[0n] | 2008 |
| *Hypoponera foreli* | MACN-bar-ins-ct 06941 | ANTPI542-15 | MF925755 | BOLD:ACZ3327 | 658[0n] | 2011 |
| *Hypoponera foreli* | MACN-bar-ins-ct 06453 | ANTI156-15 | MF925788 | BOLD:ACZ3327 | 658[0n] | 2015 |
| *Hypoponera foreli* | MACN-bar-ins-ct 06456 | ANTI159-15 | MF925751 | BOLD:ACZ3327 | 658[0n] | 2015 |
| *Hypoponera foreli* | MACN-bar-ins-ct 07053 | ANTPI654-16 | MF925844 | BOLD:ACZ3327 | 658[0n] | 2016 |
| *Hypoponera foreli* | MACN-Bar-Ins-ct 06819 | ANTPI420-15 |  |  | 0 | 1998 |
| *Hypoponera opaciceps* | MACN-bar-ins-ct 06923 | ANTPI524-15 |  |  | 0 | 2003 |
| *Hypoponera parva (**)* | MACN-bar-ins-ct 06457 | ANTI160-15 | MF925820 | BOLD:ACZ4236 | 658[0n] | 2015 |
| *Hypoponera parva (**)* | MACN-bar-ins-ct 06481 | ANTI184-15 | MF926011 | BOLD:ACZ4236 | 658[0n] | 2015 |
| *Hypoponera parva (**)* | MACN-Bar-Ins-ct 06823 | ANTPI424-15 |  |  | 0 | 1999 |
| *Hypoponera parva (**)* | MACN-Bar-Ins-ct 06850 | ANTPI451-15 |  |  | 0 | 1999 |
| *Hypoponera parva (**)* | MACN-Bar-Ins-ct 06859 | ANTPI460-15 |  |  | 0 | 1999 |
| *Hypoponera parva (**)* | MACN-bar-ins-ct 06935 | ANTPI536-15 |  |  | 0 | 2005 |
| *Hypoponera parva (**)* | MACN-bar-ins-ct 06944 | ANTPI545-15 |  |  | 0 | 2005 |
| *Hypoponera PEH01* | MACN-bar-ins-ct 06450 | ANTI153-15 | MF925746 | BOLD:ACZ4369 | 624[0n] | 2015 |
| *Hypoponera PEH02* | MACN-bar-ins-ct 06434 | ANTI137-15 | MF925808 | BOLD:ACN1914 | 658[0n] | 2015 |
| *Hypoponera PEH02* | MACN-bar-ins-ct 06439 | ANTI142-15 | MF925801 | BOLD:ACN1914 | 658[0n] | 2015 |
| *Hypoponera PEH02* | MACN-bar-ins-ct 06444 | ANTI147-15 | MF925789 | BOLD:ACN1914 | 658[0n] | 2015 |
| *Hypoponera PEH02* | MACN-bar-ins-ct 06447 | ANTI150-15 | MF925923 | BOLD:ACN1914 | 658[0n] | 2015 |
| *Hypoponera PEH02* | MACN-Bar-Ins-ct 06870 | ANTPI471-15 |  |  | 0 | 1999 |
| *Hypoponera schmalzi* | MACN-bar-ins-ct 06885 | ANTPI486-15 |  |  | 0 | 2003 |
| *Hypoponera schmalzi* | MACN-bar-ins-ct 06883 | ANTPI484-15 |  |  | 0 | 2005 |
| *Hypoponera trigona* | T3W5A2008PNIB06 | ANTPI018-10 | MF925935 | BOLD:AAU1873 | 658[1n] | 2008 |
| *Hypoponera trigona* | MACN-bar-ins-ct 06428 | ANTI131-15 | MF926047 | BOLD:AAU1873 | 632[0n] | 2015 |
| *Hypoponera trigona* | MACN-bar-ins-ct 06430 | ANTI133-15 | MF925825 | BOLD:AAU1873 | 658[0n] | 2015 |
| *Hypoponera trigona* | MACN-Bar-Ins-ct 06828 | ANTPI429-15 | MF925811 | BOLD:ACZ3162 | 658[0n] | 1999 |
| *Hypoponera trigona* | MACN-bar-ins-ct 06432 | ANTI135-15 | MF925902 | BOLD:ACZ4059 | 658[0n] | 2015 |
| *Hypoponera trigona* | MACN-bar-ins-ct 06938 | ANTPI539-15 |  |  | 0 | 2003 |
| *Hypoponera trigona* | MACN-bar-ins-ct 06951 | ANTPI552-15 |  |  | 0 | 2003 |
| *Hypoponera trigona* | T3W52008PNIB08 | ANTPI020-10 |  |  | 0 | 2008 |
| *Hypoponera trigona* | T4W12008PNIH08 | ANTPI092-10 |  |  | 0 | 2008 |
| *Labidus* | MACN-Bar-Ins-ct 00605 | INSAR129-11 |  |  | 0 | 2010 |
| *Labidus coecus* | T3S16m2009PNIC06 | ANTPI030-10 | MF925821 | BOLD:AAE5557 | 658[0n] | 2009 |
| *Labidus coecus* | T1S6a2009PNID03 | ANTPI039-10 | MF925887 | BOLD:AAU1427 | 658[0n] | 2009 |
| *Labidus coecus* | T1S8a2009PNIC02 | ANTPI026-10 | MF925839 | BOLD:AAU1427 | 658[0n] | 2009 |
| *Labidus coecus* | T3S16m2009PNIF10 | ANTPI070-10 | MF925908 | BOLD:AAU1427 | 658[0n] | 2009 |
| *Labidus coecus* | T5S12A2009PNIG01 | ANTPI073-10 | MF925992 | BOLD:AAU1427 | 658[0n] | 2009 |
| *Labidus coecus* | T5S4a2009PNIG05 | ANTPI077-10 | MF926041 | BOLD:AAU1427 | 658[0n] | 2009 |
| *Labidus coecus* | MACN-bar-ins-ct 06400 | ANTI103-15 | MF925899 | BOLD:AAU1427 | 658[0n] | 2015 |
| *Labidus coecus* | MACN-bar-ins-ct 06874 | ANTPI475-15 | MF926035 | BOLD:AAU1427 | 658[0n] | 2015 |
| *Labidus coecus* | MACN-Bar-Ins-ct 05025 | ANTPI209-13 |  |  | 0 | 2009 |
| *Labidus PEH01* | MACN-Bar-Ins-ct 02494 | INSAR635-11 |  |  | 421[0n] | 2011 |
| *Labidus praedator* | MACN-Bar-Ins-ct 02493 | INSAR634-11 | MF925979 | BOLD:ABV2652 | 658[0n] | 2011 |
| *Labidus praedator* | T3P42008PNIE10 | ANTPI058-10 |  |  | 0 | 2008 |
| *Labidus praedator* | MACN-bar-ins-ct 06397 | ANTI100-15 |  |  | 0 | 2015 |
| *Lachnomyrmex plaumanni* | MACN-Bar-Ins-ct 06789 | ANTPI390-15 |  |  | 0 | 1999 |
| *Lachnomyrmex plaumanni* | MACN-Bar-Ins-ct 06851 | ANTPI452-15 |  |  | 0 | 1999 |
| *Leptogenys iheringi(**)* | MACN-bar-ins-ct 07007 | ANTPI608-16 | MF925888 | BOLD:ADE4308 | 658[0n] | 2016 |
| *Linepithema humile* | MACN-Bar-Ins-ct 06778 | ANTPI379-15 |  |  | 470[0n] | 1999 |
| *Linepithema humile* | MACN-Bar-Ins-ct 06793 | ANTPI394-15 |  |  | 0 | 1999 |
| *Linepithema humile* | MACN-Bar-Ins-ct 06825 | ANTPI426-15 |  |  | 0 | 1999 |
| *Linepithema iniquum* | MACN-bar-ins-ct 06928 | ANTPI529-15 | MF925816 | BOLD:ACZ3624 | 649[0n] | 2011 |
| *Linepithema iniquum* | MACN-Bar-Ins-ct 06783 | ANTPI384-15 |  |  | 0 | 1999 |
| *Linepithema iniquum* | MACN-Bar-Ins-ct 06797 | ANTPI398-15 |  |  | 0 | 1999 |
| *Linepithema micans* | MACN-Bar-Ins-ct 02945 | ANTPI162-12 | MF925743 | BOLD:AAD2894 | 658[0n] | 2011 |
| *Linepithema micans* | MACN-Bar-Ins-ct 02954 | ANTPI171-12 | MF925766 | BOLD:AAD2894 | 658[0n] | 2011 |
| *Linepithema micans* | MACN-Bar-Ins-ct 02969 | ANTPI186-12 | MF926029 | BOLD:AAD2894 | 658[0n] | 2011 |
| *Linepithema micans* | MACN-bar-ins-ct 06445 | ANTI148-15 | MF925981 | BOLD:AAD2894 | 658[0n] | 2015 |
| *Linepithema micans* | MACN-Bar-Ins-ct 05040 | ANTPI266-13 |  |  | 0 | 2011 |
| *Linepithema pulex* | MACN-Bar-Ins-ct 05035 | ANTPI212-13 | MF925955 | BOLD:AAX5108 | 658[0n] | 2011 |
| *Linepithema pulex* | MACN-Bar-Ins-ct 06811 | ANTPI412-15 |  |  | 0 | 1999 |
| *Linepithema pulex* | MACN-Bar-Ins-ct 06835 | ANTPI436-15 |  |  | 0 | 1999 |
| *Linepithema pulex* | MACN-Bar-Ins-ct 06843 | ANTPI444-15 |  |  | 0 | 1999 |
| *Megalomyrmex brandaoi (**)* | MACN-Bar-Ins-ct 06840 | ANTPI441-15 | MF925994 | BOLD:ACZ3268 | 658[0n] | 1998 |
| *Megalomyrmex megadrifti (*)* | T3W52008PNIG09 | ANTPI081-10 | MF925997 | BOLD:ADB2786 | 567[0n] | 2008 |
| *Megalomyrmex megadrifti (*)* | MACN-Bar-Ins-ct 06794 | ANTPI395-15 |  |  | 0 | 1999 |
| *Megalomyrmex megadrifti (*)* | MACN-Bar-Ins-ct 06862 | ANTPI463-15 |  |  | 0 | 1999 |
| *Megalomyrmex megadrifti (*)* | MACN-bar-ins-ct 06906 | ANTPI507-15 |  |  | 0 | 2015 |
| *Megalomyrmex miri* | MACN-Bar-Ins-ct 06817 | ANTPI418-15 | MF925983 | BOLD:ACZ2861 | 658[0n] | 1999 |
| *Megalomyrmex miri* | MACN-Bar-Ins-ct 06834 | ANTPI435-15 |  |  | 0 | 1999 |
| *Mycetarotes parallelus* | MACN-bar-ins-ct 06877 | ANTPI478-15 | MF925757 | BOLD:ACZ2682 | 658[0n] | 2015 |
| *Mycocepurus smithii* | MACN-Bar-Ins-ct 02901 | ANTPI118-12 | MF926032 | BOLD:ACC4194 | 658[0n] | 2011 |
| *Mycocepurus smithii* | MACN-Bar-Ins-ct 05045 | ANTPI214-13 | MF925744 | BOLD:ACC4194 | 658[0n] | 2011 |
| *Mycocepurus smithii* | MACN-bar-ins-ct 06921 | ANTPI522-15 | MF926042 | BOLD:ACC4194 | 658[0n] | 2015 |
| *Mycocepurus smithii* | MACN-bar-ins-ct 06911 | ANTPI512-15 |  |  | 0 | 2015 |
| *Myrmelachista catharinae (**)* | MACN-bar-ins-ct 06931 | ANTPI532-15 | MF925752 | BOLD:ACZ2735 | 658[0n] | 2015 |
| *Myrmelachista catharinae (**)* | MACN-bar-ins-ct 06946 | ANTPI547-15 | MF926004 | BOLD:ACZ2735 | 658[0n] | 2015 |
| *Myrmelachista nodigera* | MACN-bar-ins-ct 06932 | ANTPI533-15 | MF925911 | BOLD:ACZ2726 | 658[0n] | 2005 |
| *Myrmelachista nodigera* | MACN-bar-ins-ct 06913 | ANTPI514-15 |  |  | 0 | 2005 |
| *Myrmicocrypta foreli* | MACN-Bar-Ins-ct 06854 | ANTPI455-15 |  |  | 0 | 1999 |
| *Myrmicocrypta foreli* | MACN-Bar-Ins-ct 02935 | ANTPI152-12 |  |  | 0 | 2011 |
| *Neivamyrmex* | MACN-bar-ins-ct 06470 | ANTI173-15 |  | BOLD:ACZ3964 | 658[0n] | 2015 |
| *Neivamyrmex angustinodis (A)* | MACN-Bar-Ins-ct 06808 | ANTPI409-15 | MF925880 | BOLD:ACZ3084 | 658[0n] | 1999 |
| *Neivamyrmex angustinodis (A)* | MACN-Bar-Ins-ct 06780 | ANTPI381-15 |  |  | 0 | 1999 |
| *Neivamyrmex punctaticeps* | MACN-Bar-Ins-ct 05034 | ANTPI211-13 | MF926006 | BOLD:ACM2493 | 658[0n] | 2011 |
| *Neivamyrmex punctaticeps* | MACN-Bar-Ins-ct 05038 | ANTPI213-13 | MF925828 | BOLD:ACM2493 | 658[0n] | 2011 |
| *Neivamyrmex punctaticeps* | MACN-bar-ins-ct 06419 | ANTI122-15 | MF925840 | BOLD:ACM2493 | 658[0n] | 2015 |
| *Neivamyrmex punctaticeps* | MACN-bar-ins-ct 06436 | ANTI139-15 | MF925857 | BOLD:ACM2493 | 658[0n] | 2015 |
| *Neivamyrmex punctaticeps* | MACN-bar-ins-ct 06902 | ANTPI503-15 | MF925774 | BOLD:ACM2493 | 658[0n] | 2015 |
| *Neoponera* | MACN-Bar-Ins-ct 02572 | INSAR745-11 |  | BOLD:ABV2663 | 658[0n] | 2011 |
| *Neoponera* | MACN-Bar-Ins-ct 02564 | INSAR738-11 |  | BOLD:ABV2684 | 658[0n] | 2011 |
| *Neoponera bactronica (**)* | MACN-bar-ins-ct 06396 | ANTI099-15 | MF925845 | BOLD:AAW5111 | 658[0n] | 2015 |
| *Neoponera crenata* | MACN-Bar-Ins-ct 06809 | ANTPI410-15 | MF925832 | BOLD:ABV2684 | 658[0n] | 1999 |
| *Neoponera crenata* | MACN-bar-ins-ct 06398 | ANTI101-15 | MF925804 | BOLD:ACX7584 | 658[0n] | 2015 |
| *Neoponera crenata* | MACN-bar-ins-ct 06416 | ANTI119-15 | MF925895 | BOLD:ACX7584 | 658[0n] | 2015 |
| *Neoponera crenata* | MACN-Bar-Ins-ct 06779 | ANTPI380-15 |  |  | 0 | 1999 |
| *Neoponera crenata* | MACN-bar-ins-ct 06891 | ANTPI492-15 |  |  | 0 | 2005 |
| *Neoponera crenata* | MACN-bar-ins-ct 06943 | ANTPI544-15 |  |  | 0 | 2005 |
| *Neoponera curvinodis (**)* | MACN-bar-ins-ct 06407 | ANTI110-15 | MF925818 | BOLD:AAW5111 | 658[0n] | 2015 |
| *Neoponera fiebrigi (A)* | MACN-bar-ins-ct 06401 | ANTI104-15 | MF925945 | BOLD:ACN1772 | 658[0n] | 2015 |
| *Neoponera moesta* | MACN-Bar-Ins-ct 05145 | ANTPI254-13 | MF925968 | BOLD:ACM2898 | 658[0n] | 2011 |
| *Neoponera moesta* | MACN-bar-ins-ct 06414 | ANTI117-15 | MF925884 | BOLD:ACM2898 | 658[0n] | 2015 |
| *Neoponera obscuricornis (*)* | MACN-bar-ins-ct 07017 | ANTPI618-16 | MF925964 | BOLD:AAZ3349 | 613[0n] | 2015 |
| *Neoponera obscuricornis (*)* | MACN-bar-ins-ct 07038 | ANTPI639-16 | MF926036 | BOLD:AAZ3349 | 658[0n] | 2015 |
| *Neoponera obscuricornis (*)* | MACN-bar-ins-ct 07054 | ANTPI655-16 | MF925794 | BOLD:AAZ3349 | 619[0n] | 2016 |
| *Neoponera obscuricornis (*)* | MACN-bar-ins-ct 06435 | ANTI138-15 |  |  | 151[0n] | 2015 |
| *Neoponera obscuricornis (*)* | MACN-bar-ins-ct 06440 | ANTI143-15 |  |  | 144[1n] | 2015 |
| *Neoponera verenae (**)* | MACN-bar-ins-ct 07061 | ANTPI662-16 | MF925961 | BOLD:ACN0470 | 658[0n] | 2011 |
| *Neoponera villosa* | MACN-Bar-Ins-ct 05121 | ANTPI242-13 | MF925837 | BOLD:AAZ7290 | 658[0n] | 2011 |
| *Neoponera villosa* | MACN-Bar-Ins-ct 05138 | ANTPI247-13 | MF925948 | BOLD:AAZ7290 | 658[0n] | 2011 |
| *Neoponera villosa* | MACN-bar-ins-ct 06404 | ANTI107-15 | MF925742 | BOLD:AAZ7290 | 658[0n] | 2015 |
| *Nesomyrmex asper* | MACN-bar-ins-ct 06876 | ANTPI477-15 | MF925848 | BOLD:ACZ2681 | 658[0n] | 2015 |
| *Nesomyrmex asper* | MACN-bar-ins-ct 06892 | ANTPI493-15 | MF926039 | BOLD:ACZ2681 | 658[0n] | 2015 |
| *Nesomyrmex asper* | MACN-bar-ins-ct 06934 | ANTPI535-15 | MF926031 | BOLD:ACZ2681 | 658[0n] | 2015 |
| *Nesomyrmex tonsuratus (**)* | MACN-bar-ins-ct 06909 | ANTPI510-15 | MF925803 | BOLD:ACZ2734 | 658[0n] | 2015 |
| *Nylanderia fulva* | MACN-bar-ins-ct 06465 | ANTI168-15 | MF925786 | BOLD:ACZ3937 | 658[0n] | 2015 |
| *Nylanderia fulva* | MACN-Bar-Ins-ct 05046 | ANTPI215-13 |  |  | 0 | 2011 |
| *Nylanderia fulva* | MACN-Bar-Ins-ct 05050 | ANTPI218-13 |  |  | 0 | 2011 |
| *Nylanderia fulva* | MACN-Bar-Ins-ct 05060 | ANTPI221-13 |  |  | 0 | 2011 |
| *Nylanderia fulva* | MACN-bar-ins-ct 06880 | ANTPI481-15 |  |  | 0 | 2011 |
| *Nylanderia fulva* | MACN-bar-ins-ct 06927 | ANTPI528-15 |  |  | 0 | 2011 |
| *Nylanderia fulva* | MACN-bar-ins-ct 06942 | ANTPI543-15 |  |  | 0 | 2011 |
| *Nylanderia PEH01* | MACN-Bar-Ins-ct 05047 | ANTPI216-13 | MF925807 | BOLD:ACM2582 | 658[0n] | 2011 |
| *Nylanderia PEH01* | MACN-Bar-Ins-ct 06810 | ANTPI411-15 |  |  | 0 | 1999 |
| *Nylanderia PEH01* | MACN-Bar-Ins-ct 05052 | ANTPI267-13 |  |  | 0 | 2008 |
| *Nylanderia PEH01* | MACN-Bar-Ins-ct 05062 | ANTPI223-13 |  |  | 0 | 2011 |
| *Nylanderia PEH02* | MACN-Bar-Ins-ct 05049 | ANTPI217-13 | MF925872 | BOLD:ACM2581 | 658[0n] | 2011 |
| *Nylanderia PEH02* | MACN-bar-ins-ct 06485 | ANTI188-15 | MF925931 | BOLD:ACM2581 | 658[0n] | 2015 |
| *Nylanderia PEH03* | T1S12m2009PNIC04 | ANTPI028-10 |  |  | 491[8n] | 2009 |
| *Octostruma balzani* | MACN-Bar-Ins-ct 06847 | ANTPI448-15 |  |  | 0 | 1999 |
| *Octostruma balzani* | MACN-Bar-Ins-ct 06866 | ANTPI467-15 |  |  | 0 | 1999 |
| *Octostruma balzani* | T1W12008PNID08 | ANTPI044-10 |  |  | 0 | 2008 |
| *Octostruma balzani* | MACN-bar-ins-ct 06947 | ANTPI548-15 |  |  | 0 | 2015 |
| *Octostruma iheringi* | T6S6a2009PNIG07 | ANTPI079-10 | MF925942 | BOLD:AAX0010 | 658[0n] | 2009 |
| *Octostruma iheringi* | T6S8a2009PNIE09 | ANTPI057-10 |  |  | 0 | 2009 |
| *Octostruma PEH01* | MACN-Bar-Ins-ct 06853 | ANTPI454-15 | MF926023 | BOLD:ACZ2651 | 658[0n] | 1998 |
| *Octostruma PEH01* | MACN-Bar-Ins-ct 06827 | ANTPI428-15 |  |  | 0 | 1998 |
| *Octostruma PEH01* | MACN-Bar-Ins-ct 06800 | ANTPI401-15 |  |  | 0 | 1999 |
| *Odontomachus chelifer* | T6P52008PNID10 | ANTPI046-10 | MF925976 | BOLD:AAV3356 | 658[0n] | 2008 |
| *Odontomachus chelifer* | MACN-Bar-Ins-ct 05137 | ANTPI246-13 | MF925881 | BOLD:AAV3356 | 658[0n] | 2011 |
| *Odontomachus chelifer* | MACN-Bar-Ins-ct 05141 | ANTPI250-13 | MF925876 | BOLD:AAV3356 | 658[0n] | 2011 |
| *Odontomachus chelifer* | MACN-bar-ins-ct 06399 | ANTI102-15 | MF926046 | BOLD:AAV3356 | 658[0n] | 2015 |
| *Odontomachus chelifer* | MACN-bar-ins-ct 06417 | ANTI120-15 | MF925784 | BOLD:AAV3356 | 658[0n] | 2015 |
| *Odontomachus meinerti* | T2W52008PNIH03 | ANTPI087-10 | MF925903 | BOLD:AAX0128 | 658[0n] | 2008 |
| *Odontomachus meinerti* | MACN-Bar-Ins-ct 05059 | ANTPI220-13 | MF925996 | BOLD:AAX0128 | 658[0n] | 2011 |
| *Odontomachus meinerti* | MACN-Bar-Ins-ct 05061 | ANTPI222-13 | MF925991 | BOLD:AAX0128 | 658[0n] | 2011 |
| *Odontomachus meinerti* | MACN-bar-ins-ct 06418 | ANTI121-15 | MF926012 | BOLD:AAX0128 | 658[0n] | 2015 |
| *Odontomachus meinerti* | MACN-bar-ins-ct 06421 | ANTI124-15 | MF925878 | BOLD:AAX0128 | 658[0n] | 2015 |
| *Odontomachus meinerti* | MACN-bar-ins-ct 06423 | ANTI126-15 | MF925910 | BOLD:AAX0128 | 658[0n] | 2015 |
| *Odontomachus meinerti* | MACN-bar-ins-ct 07018 | ANTPI619-16 | MF925779 | BOLD:AAX0128 | 658[0n] | 2016 |
| *Odontomachus meinerti* | MACN-bar-ins-ct 07060 | ANTPI661-16 | MF926002 | BOLD:AAX0128 | 658[0n] | 2016 |
| *Odontomachus meinerti* | MACN-Bar-Ins-ct 05067 | ANTPI227-13 | MF926045 | BOLD:ACM2983 | 658[0n] | 2008 |
| *Odontomachus meinerti* | MACN-bar-ins-ct 06402 | ANTI105-15 | MF925977 | BOLD:ACM2983 | 658[0n] | 2015 |
| *Odontomachus meinerti* | MACN-Bar-Ins-ct 05071 | ANTPI269-13 |  |  | 0 | 2008 |
| *Odontomachus meinerti* | MACN-Bar-Ins-ct 05065 | ANTPI225-13 |  |  | 0 | 2009 |
| *Pachycondyla constricticeps* | MACN-bar-ins-ct 06409 | ANTI112-15 |  |  | 0 | 2008 |
| *Pachycondyla harpax* | T3S6m2009PNIC09 | ANTPI033-10 | MF925954 | BOLD:AAU1874 | 658[0n] | 2009 |
| *Pachycondyla PEH01* | MACN-bar-ins-ct 06411 | ANTI114-15 | MF925894 | BOLD:ACZ4044 | 658[0n] | 2015 |
| *Pachycondyla striata* | MACN-Bar-Ins-ct 02937 | ANTPI154-12 | MF926017 | BOLD:AAU1872 | 658[0n] | 2008 |
| *Pachycondyla striata* | MACN-Bar-Ins-ct 05051 | ANTPI219-13 | MF925835 | BOLD:AAU1872 | 658[0n] | 2008 |
| *Pachycondyla striata* | T1P12008PNIE02 | ANTPI050-10 | MF925916 | BOLD:AAU1872 | 658[1n] | 2008 |
| *Pachycondyla striata* | T3P42008PNIH07 | ANTPI091-10 | MF925918 | BOLD:AAU1872 | 658[0n] | 2008 |
| *Pachycondyla striata* | MACN-Bar-Ins-ct 05729 | INSAR1393-15 |  |  | 0 | 2014 |
| *Pachycondyla striata* | MACN-Bar-Ins-ct 05735 | INSAR1399-15 |  |  | 0 | 2014 |
| *Pheidole* | MACN-bar-ins-ct 06948 | ANTPI549-15 |  | BOLD:ACM2643 | 634[0n] | 2011 |
| *Pheidole* | MACN-Bar-Ins-ct 05119 | ANTPI278-13 |  |  | 0 | 2008 |
| *Pheidole* | T1W12008PNIB09 | ANTPI021-10 |  |  | 0 | 2008 |
| *Pheidole* | T1W12008PNIB11 | ANTPI023-10 |  |  | 0 | 2008 |
| *Pheidole* | T3W52008PNIF02 | ANTPI062-10 |  |  | 0 | 2008 |
| *Pheidole* | T3W52008PNIH11 | ANTPI095-10 |  |  | 0 | 2008 |
| *Pheidole* | M22009PNIA10 | ANTPI010-10 |  |  | 0 | 2009 |
| *Pheidole* | T1S16M2009PNIA03 | ANTPI003-10 |  |  | 591[2n] | 2009 |
| *Pheidole* | MACN-Bar-Ins-ct 02889 | ANTPI106-12 |  |  | 0 | 2011 |
| *Pheidole* | MACN-Bar-Ins-ct 02966 | ANTPI183-12 |  |  | 0 | 2011 |
| *Pheidole* | MACN-Bar-Ins-ct 05116 | ANTPI241-13 |  |  | 0 | 2011 |
| *Pheidole alpinensis (*)* | MACN-Bar-Ins-ct 02899 | ANTPI116-12 | MF925760 | BOLD:ACC4235 | 658[0n] | 2011 |
| *Pheidole alpinensis (*)* | MACN-Bar-Ins-ct 02913 | ANTPI130-12 |  |  | 0 | 2011 |
| *Pheidole cl. dinophila (**)* | MACN-bar-ins-ct 06873 | ANTPI474-15 |  |  | 0 | 2005 |
| *Pheidole cl. dinophila (**)* | MACN-bar-ins-ct 06886 | ANTPI487-15 |  |  | 0 | 2005 |
| *Pheidole cl. dinophila (**)* | MACN-bar-ins-ct 06896 | ANTPI497-15 |  |  | 0 | 2008 |
| *Pheidole cl. dinophila (**)* | MACN-bar-ins-ct 06920 | ANTPI521-15 |  |  | 0 | 2008 |
| *Pheidole fimbriata* | T4S18A2009PNIA11 | ANTPI011-10 | MF925883 | BOLD:AAU2524 | 658[0n] | 2009 |
| *Pheidole fimbriata* | T5S4m2009PNIC01 | ANTPI025-10 | MF925740 | BOLD:AAU2524 | 658[0n] | 2009 |
| *Pheidole fimbriata* | MACN-Bar-Ins-ct 05066 | ANTPI226-13 | MF925932 | BOLD:ACM2608 | 658[0n] | 2011 |
| *Pheidole gertrudae* | MACN-Bar-Ins-ct 05104 | ANTPI282-13 |  |  | 0 | 2011 |
| *Pheidole gertrudae* | MACN-Bar-Ins-ct 05110 | ANTPI237-13 |  |  | 0 | 2011 |
| *Pheidole mosenopsis* | MACN-bar-ins-ct 06898 | ANTPI499-15 | MF925882 | BOLD:AAU2525 | 658[0n] | 2005 |
| *Pheidole mosenopsis* | T2S4M2009PNIA05 | ANTPI005-10 | MF925796 | BOLD:AAU2525 | 658[0n] | 2009 |
| *Pheidole mosenopsis* | MACN-bar-ins-ct 06875 | ANTPI476-15 |  |  | 0 | 2005 |
| *Pheidole mosenopsis* | MACN-bar-ins-ct 06890 | ANTPI491-15 |  |  | 0 | 2005 |
| *Pheidole mosenopsis* | MACN-bar-ins-ct 06893 | ANTPI494-15 |  |  | 0 | 2005 |
| *Pheidole mosenopsis* | MACN-bar-ins-ct 06919 | ANTPI520-15 |  |  | 168[0n] | 2005 |
| *Pheidole mosenopsis* | T1W12008PNIB04 | ANTPI016-10 |  |  | 0 | 2008 |
| *Pheidole obscurithorax* | MACN-bar-ins-ct 06956 | ANTPI557-15 | MF926030 | BOLD:ACZ4199 | 658[0n] | 2011 |
| *Pheidole PEH01* | MACN-Bar-Ins-ct 02883 | ANTPI100-12 | MF926010 | BOLD:ACC4288 | 658[0n] | 2011 |
| *Pheidole PEH01* | MACN-Bar-Ins-ct 02891 | ANTPI108-12 | MF925873 | BOLD:ACC4288 | 658[0n] | 2011 |
| *Pheidole PEH01* | MACN-Bar-Ins-ct 02909 | ANTPI126-12 | MF925928 | BOLD:ACC4288 | 658[0n] | 2011 |
| *Pheidole PEH01* | MACN-Bar-Ins-ct 02923 | ANTPI140-12 | MF926005 | BOLD:ACC4288 | 658[0n] | 2011 |
| *Pheidole PEH01* | MACN-Bar-Ins-ct 02938 | ANTPI155-12 | MF925850 | BOLD:ACC4288 | 658[0n] | 2011 |
| *Pheidole PEH01* | MACN-Bar-Ins-ct 02941 | ANTPI158-12 | MF926033 | BOLD:ACC4288 | 658[0n] | 2011 |
| *Pheidole PEH01* | MACN-Bar-Ins-ct 02956 | ANTPI173-12 | MF925938 | BOLD:ACC4288 | 658[0n] | 2011 |
| *Pheidole PEH02* | T1S12A2009PNIA08 | ANTPI008-10 | MF925953 | BOLD:AAU4344 | 658[0n] | 2009 |
| *Pheidole PEH02* | T5S4m2009PNIC03 | ANTPI027-10 | MF925764 | BOLD:AAU4344 | 658[0n] | 2009 |
| *Pheidole PEH02* | T3S16a2008PNIC12 | ANTPI036-10 | MF926024 | BOLD:AAU4345 | 658[0n] | 2008 |
| *Pheidole PEH02* | MACN-Bar-Ins-ct 02893 | ANTPI110-12 | MF926043 | BOLD:AAU4345 | 658[0n] | 2011 |
| *Pheidole PEH02* | MACN-Bar-Ins-ct 02943 | ANTPI160-12 | MF925967 | BOLD:AAU4345 | 658[0n] | 2011 |
| *Pheidole PEH02* | MACN-Bar-Ins-ct 02965 | ANTPI182-12 | MF925798 | BOLD:AAU4345 | 658[0n] | 2011 |
| *Pheidole PEH02* | MACN-Bar-Ins-ct 02971 | ANTPI188-12 | MF925841 | BOLD:AAU4345 | 658[0n] | 2011 |
| *Pheidole PEH02* | T3S16m2009PNIC05 | ANTPI029-10 |  |  | 0 | 2009 |
| *Pheidole PEH02* | MACN-Bar-Ins-ct 02879 | ANTPI096-12 |  |  | 0 | 2011 |
| *Pheidole PEH02* | MACN-Bar-Ins-ct 02928 | ANTPI145-12 | XXXX |  | 0 | 2011 |
| *Pheidole PEH02* | MACN-Bar-Ins-ct 02952 | ANTPI169-12 |  |  | 658[0n] | 2011 |
| *Pheidole PEH03* | MACN-Bar-Ins-ct 02887 | ANTPI104-12 | MF925913 | BOLD:ACC4180 | 658[0n] | 2011 |
| *Pheidole PEH03* | MACN-Bar-Ins-ct 02905 | ANTPI122-12 | MF925907 | BOLD:ACC4180 | 658[0n] | 2011 |
| *Pheidole PEH03* | MACN-Bar-Ins-ct 05080 | ANTPI229-13 | MF925809 | BOLD:ACC4180 | 658[0n] | 2011 |
| *Pheidole PEH04* | MACN-Bar-Ins-ct 02925 | ANTPI142-12 | MF925861 | BOLD:ACC4354 | 658[0n] | 2011 |
| *Pheidole PEH04* | MACN-Bar-Ins-ct 02930 | ANTPI147-12 | MF925925 | BOLD:ACC4354 | 658[0n] | 2011 |
| *Pheidole PEH04* | MACN-Bar-Ins-ct 05112 | ANTPI238-13 | MF925949 | BOLD:ACC4354 | 658[0n] | 2011 |
| *Pheidole PEH04* | MACN-Bar-Ins-ct 05154 | ANTPI262-13 | MF925957 | BOLD:ACC4354 | 658[0n] | 2011 |
| *Pheidole PEH05* | MACN-Bar-Ins-ct 731 | INSAR540-11 | MF925984 | BOLD:AAZ4402 | 658[0n] | 2010 |
| *Pheidole PEH06* | MACN-Bar-Ins-ct 02962 | ANTPI179-12 | MF925860 | BOLD:AAP9302 | 658[0n] | 2011 |
| *Pheidole PEH09* | MACN-Bar-Ins-ct 02907 | ANTPI124-12 | MF925823 | BOLD:AAL5917 | 658[0n] | 2011 |
| *Pheidole PEH09* | MACN-Bar-Ins-ct 02921 | ANTPI138-12 | MF925863 | BOLD:AAL5917 | 658[0n] | 2011 |
| *Pheidole PEH10* | T1S10M2009PNIA01 | ANTPI001-10 |  |  | 0 | 2009 |
| *Pheidole PEH11* | MACN-Bar-Ins-ct 02902 | ANTPI119-12 |  |  | 0 | 2011 |
| *Pheidole PEH12* | MACN-bar-ins-ct 06881 | ANTPI482-15 |  |  | 0 | 2005 |
| *Pheidole PEH12* | MACN-bar-ins-ct 06966 | ANTPI567-15 |  |  | 0 | 2005 |
| *Pheidole PEH12* | MACN-Bar-Ins-ct 02932 | ANTPI149-12 |  |  | 0 | 2011 |
| *Pheidole PEH12* | MACN-Bar-Ins-ct 02960 | ANTPI177-12 |  |  | 0 | 2011 |
| *Pheidole rudigenis* | MACN-bar-ins-ct 06916 | ANTPI517-15 |  |  | 0 | 2003 |
| *Pheidole rugatula* | MACN-Bar-Ins-ct 02885 | ANTPI102-12 | MF925830 | BOLD:ACC4289 | 658[0n] | 2011 |
| *Pheidole rugatula* | MACN-Bar-Ins-ct 02934 | ANTPI151-12 | MF925852 | BOLD:ACC4289 | 658[0n] | 2011 |
| *Pheidole rugatula* | MACN-Bar-Ins-ct 05070 | ANTPI228-13 | MF925775 | BOLD:ACC4289 | 658[0n] | 2011 |
| *Pheidole rugatula* | MACN-Bar-Ins-ct 05122 | ANTPI276-13 | MF925859 | BOLD:ACC4289 | 658[0n] | 2011 |
| *Pheidole sigillata (**)* | MACN-Bar-Ins-ct 02897 | ANTPI114-12 | MF925769 | BOLD:ACC4420 | 658[0n] | 2011 |
| *Pheidole sigillata (**)* | MACN-Bar-Ins-ct 02917 | ANTPI134-12 | MF925754 | BOLD:ACC4420 | 658[0n] | 2011 |
| *Pheidole sigillata (**)* | MACN-Bar-Ins-ct 02958 | ANTPI175-12 | MF925875 | BOLD:ACC4420 | 658[0n] | 2011 |
| *Pheidole sigillata (**)* | MACN-Bar-Ins-ct 05081 | ANTPI230-13 | MF925842 | BOLD:ACC4420 | 658[0n] | 2011 |
| *Pheidole sigillata (**)* | MACN-Bar-Ins-ct 05088 | ANTPI233-13 | MF926027 | BOLD:ACC4420 | 658[0n] | 2011 |
| *Pheidole sigillata (**)* | T2S2A2009PNIA04 | ANTPI004-10 |  |  | 0 | 2009 |
| *Pheidole sigillata (**)* | MACN-Bar-Ins-ct 02936 | ANTPI153-12 |  |  | 0 | 2011 |
| *Pheidole subarmata* | T1S8M2009PNIA02 | ANTPI002-10 | MF925919 | BOLD:AAU4342 | 658[2n] | 2009 |
| *Pheidole subarmata* | T3S10A2009PNIG12 | ANTPI084-10 | MF926015 | BOLD:AAU4342 | 658[0n] | 2009 |
| *Pheidole subarmata* | T3S14M2009PNIA06 | ANTPI006-10 | MF925773 | BOLD:AAU4342 | 658[0n] | 2009 |
| *Pheidole subarmata* | MACN-Bar-Ins-ct 02939 | ANTPI156-12 | MF925999 | BOLD:AAU4342 | 658[0n] | 2011 |
| *Pheidole subarmata* | MACN-Bar-Ins-ct 02963 | ANTPI180-12 | MF925930 | BOLD:AAU4342 | 649[0n] | 2011 |
| *Pheidole subarmata* | T3S2A2009PNIA07 | ANTPI007-10 | MF925781 | BOLD:ACM2643 | 658[0n] | 2009 |
| *Pheidole subarmata* | T3S2a2009PNIE01 | ANTPI049-10 | MF925741 | BOLD:ACM2643 | 658[0n] | 2009 |
| *Pheidole subarmata* | T3S4a2009PNIC07 | ANTPI031-10 | MF925958 | BOLD:ACM2643 | 658[0n] | 2009 |
| *Pheidole subarmata* | T4S16A2009PNIH04 | ANTPI088-10 | MF925870 | BOLD:ACM2643 | 658[0n] | 2009 |
| *Pheidole subarmata* | T4S4M2009PNIA09 | ANTPI009-10 | MF925874 | BOLD:ACM2643 | 658[0n] | 2009 |
| *Pheidole subarmata* | MACN-Bar-Ins-ct 02881 | ANTPI098-12 | MF925974 | BOLD:ACM2643 | 658[0n] | 2011 |
| *Pheidole subarmata* | MACN-Bar-Ins-ct 02895 | ANTPI112-12 | MF925834 | BOLD:ACM2643 | 658[0n] | 2011 |
| *Pheidole subarmata* | MACN-Bar-Ins-ct 02903 | ANTPI120-12 | MF925956 | BOLD:ACM2643 | 658[0n] | 2011 |
| *Pheidole subarmata* | MACN-Bar-Ins-ct 05064 | ANTPI224-13 | MF925856 | BOLD:ACM2643 | 658[0n] | 2011 |
| *Pheidole subarmata* | MACN-Bar-Ins-ct 05114 | ANTPI239-13 | MF925906 | BOLD:ACM2643 | 658[0n] | 2011 |
| *Pheidole subarmata* | T1W12008PNIB03 | ANTPI015-10 |  |  | 0 | 2008 |
| *Pheidole subarmata* | T1S18M2009PNIG06 | ANTPI078-10 |  |  | 0 | 2009 |
| *Pheidole subarmata* | T3S10a2009PNIF09 | ANTPI069-10 |  |  | 0 | 2009 |
| *Pheidole subarmata* | T5S10m2009PNIE06 | ANTPI054-10 |  |  | 0 | 2009 |
| *Pheidole subarmata* | T5S10m2009PNIE08 | ANTPI056-10 |  |  | 0 | 2009 |
| *Pheidole subarmata* | MACN-Bar-Ins-ct 02919 | ANTPI136-12 |  |  | 0 | 2011 |
| *Platythyrea pilosula (**)* | MACN-bar-ins-ct 06459 | ANTI162-15 | MF925988 | BOLD:ACY5292 | 658[0n] | 2015 |
| *Pogonomyrmex naegelii* | MACN-bar-ins-ct 06469 | ANTI172-15 | MF925762 | BOLD:AAL0334 | 658[0n] | 2015 |
| *Pogonomyrmex naegelii* | MACN-bar-ins-ct 06915 | ANTPI516-15 |  |  | 0 | 2015 |
| *Procryptocerus adlerzi (**)* | MACN-Bar-Ins-ct 05142 | ANTPI251-13 | MF925971 | BOLD:ACM2934 | 658[0n] | 2011 |
| *Procryptocerus hylaeus* | MACN-bar-ins-ct 06410 | ANTI113-15 | MF925854 | BOLD:ACQ7782 | 658[0n] | 2015 |
| *Procryptocerus hylaeus* | MACN-bar-ins-ct 06412 | ANTI115-15 | MF925950 | BOLD:ACQ7782 | 658[0n] | 2015 |
| *Procryptocerus hylaeus* | MACN-bar-ins-ct 06950 | ANTPI551-15 | MF925973 | BOLD:ACQ7782 | 658[0n] | 2015 |
| *Procryptocerus hylaeus* | MACN-bar-ins-ct 06884 | ANTPI485-15 |  |  | 0 | 2015 |
| *Procryptocerus regularis* | MACN-Bar-Ins-ct 05147 | ANTPI255-13 | MF925814 | BOLD:ACM2933 | 658[0n] | 2011 |
| *Pseudomyrmex cf. pupa (**)* | MACN-Bar-Ins-ct 05150 | ANTPI258-13 |  |  | 0 | 2011 |
| *Pseudomyrmex cf. pupa (**)* | MACN-bar-ins-ct 06424 | ANTI127-15 |  |  | 0 | 2015 |
| *Pseudomyrmex gracilis* | MACN-Bar-Ins-ct 02926 | ANTPI143-12 | MF925776 | BOLD:ACC4398 | 658[0n] | 2011 |
| *Pseudomyrmex gracilis* | MACN-Bar-Ins-ct 02957 | ANTPI174-12 |  |  | 0 | 2011 |
| *Pseudomyrmex gracilis* | MACN-Bar-Ins-ct 05087 | ANTPI232-13 |  |  | 0 | 2011 |
| *Pseudomyrmex gracilis* | MACN-Bar-Ins-ct 05148 | ANTPI256-13 |  |  | 0 | 2011 |
| *Pseudomyrmex gracilis* | MACN-bar-ins-ct 06406 | ANTI109-15 |  |  | 0 | 2015 |
| *Pseudomyrmex PEH01* | MACN-Bar-Ins-ct 05139 | ANTPI248-13 | MF925912 | BOLD:ACM3001 | 658[0n] | 2011 |
| *Pseudomyrmex PEH02* | MACN-bar-ins-ct 06986 | ANTPI587-16 | MF925810 | BOLD:ACM9913 | 591[1n] | 2015 |
| *Pseudomyrmex PEH03* | MACN-bar-ins-ct 06963 | ANTPI564-15 |  |  | 0 | 1998 |
| *Pseudomyrmex phyllophilus* | MACN-bar-ins-ct 06962 | ANTPI563-15 |  |  | 0 | 1999 |
| *Pseudomyrmex schuppi* | MACN-bar-ins-ct 06458 | ANTI161-15 | MF925765 | BOLD:ACV2863 | 658[0n] | 2015 |
| *Pseudomyrmex simplex* | MACN-bar-ins-ct 06889 | ANTPI490-15 | MF925966 | BOLD:ACZ3034 | 658[0n] | 2015 |
| *Pseudomyrmex termitarius (A)* | MACN-bar-ins-ct 06908 | ANTPI509-15 |  |  | 0 | 2015 |
| *Pseudomyrmex urbanus (A)* | MACN-Bar-Ins-ct 02911 | ANTPI128-12 |  |  | 0 | 2011 |
| *Rogeria PEH01* | MACN-Bar-Ins-ct 06856 | ANTPI457-15 |  |  | 0 | 1999 |
| *Rogeria scobinata* | MACN-Bar-Ins-ct 06786 | ANTPI387-15 |  |  | 0 | 1999 |
| *Rogeria scobinata* | MACN-Bar-Ins-ct 06806 | ANTPI407-15 |  |  | 0 | 1999 |
| *Rogeria scobinata* | MACN-Bar-Ins-ct 06816 | ANTPI417-15 |  |  | 0 | 1999 |
| *Rogeria scobinata* | MACN-Bar-Ins-ct 06845 | ANTPI446-15 |  |  | 0 | 1999 |
| *Solenopsis cf. picea (*)* | T1W32008PNIB07 | ANTPI019-10 |  |  | 498[4n] | 2008 |
| *Solenopsis cf. picea (*)* | T3W32008PNID02 | ANTPI038-10 |  |  | 0 | 2008 |
| *Solenopsis helena (*)* | MACN-Bar-Ins-ct 02900 | ANTPI117-12 | MF925753 | BOLD:ACC4227 | 658[0n] | 2011 |
| *Solenopsis helena (*)* | MACN-Bar-Ins-ct 02918 | ANTPI135-12 | MF925941 | BOLD:ACC4227 | 658[0n] | 2011 |
| *Solenopsis helena (*)* | MACN-bar-ins-ct 06917 | ANTPI518-15 |  |  | 0 | 2015 |
| *Solenopsis iheringi (**)* | MACN-Bar-Ins-ct 05105 | ANTPI235-13 | MF925871 | BOLD:AAE8643 | 658[0n] | 2009 |
| *Solenopsis iheringi (**)* | T1W12008PNIG08 | ANTPI080-10 |  |  | 0 | 2008 |
| *Solenopsis iheringi (**)* | T1W32008PNIB01 | ANTPI013-10 |  |  | 0 | 2008 |
| *Solenopsis iheringi (**)* | T1W32008PNIB02 | ANTPI014-10 |  |  | 0 | 2008 |
| *Solenopsis iheringi (**)* | MACN-bar-ins-ct 06899 | ANTPI500-15 |  |  | 0 | 2015 |
| *Solenopsis PEH01* | MACN-bar-ins-ct 06955 | ANTPI556-15 | MF925826 | BOLD:ACW4714 | 627[0n] | 2015 |
| *Solenopsis PEH02* | MACN-bar-ins-ct 06933 | ANTPI534-15 | MF925866 | BOLD:ACZ3333 | 658[0n] | 2015 |
| *Solenopsis PEH03* | T3W52008PNIA12 | ANTPI012-10 |  |  | 0 | 2008 |
| *Solenopsis PEH04* | T1W32008PNIB10 | ANTPI022-10 |  |  | 658[1n] | 2008 |
| *Solenopsis PEH04* | MACN-Bar-Ins-ct 02947 | ANTPI164-12 |  |  | 0 | 2011 |
| *Solenopsis PEH04* | MACN-Bar-Ins-ct 02967 | ANTPI184-12 |  |  | 0 | 2011 |
| *Solenopsis PEH04* | MACN-Bar-Ins-ct 05134 | ANTPI279-13 |  |  | 0 | 2011 |
| *Solenopsis PEH06* | MACN-Bar-Ins-ct 05107 | ANTPI236-13 | MF925759 | BOLD:ACM2874 | 658[0n] | 2011 |
| *Solenopsis PEH06* | MACN-Bar-Ins-ct 05115 | ANTPI240-13 | MF925915 | BOLD:ACM2874 | 658[0n] | 2011 |
| *Solenopsis PEH06* | MACN-Bar-Ins-ct 05083 | ANTPI272-13 |  |  | 0 | 2011 |
| *Solenopsis PEH06* | MACN-Bar-Ins-ct 05086 | ANTPI231-13 |  |  | 0 | 2011 |
| *Solenopsis PEH06* | MACN-Bar-Ins-ct 05130 | ANTPI245-13 |  |  | 0 | 2011 |
| *Solenopsis PEH06* | MACN-Bar-Ins-ct 05131 | ANTPI281-13 |  |  | 0 | 2011 |
| *Solenopsis PEH06* | MACN-Bar-Ins-ct 05146 | ANTPI283-13 |  |  | 0 | 2011 |
| *Solenopsis PEH07* | T2S20m2009PNIF03 | ANTPI063-10 |  |  | 0 | 2009 |
| *Solenopsis PEH09* | T1W32008PNIB12 | ANTPI024-10 |  |  | 0 | 2008 |
| *Solenopsis richteri (A)* | MACN-bar-ins-ct 06467 | ANTI170-15 | MF925833 | BOLD:ABV0845 | 658[0n] | 2015 |
| *Solenopsis richteri (A)* | MACN-bar-ins-ct 06925 | ANTPI526-15 | MF925986 | BOLD:ABV0845 | 658[0n] | 2015 |
| *Solenopsis richteri (A)* | MACN-bar-ins-ct 06930 | ANTPI531-15 | MF926001 | BOLD:ABV0845 | 658[0n] | 2015 |
| *Solenopsis richteri (A)* | MACN-bar-ins-ct 06978 | ANTPI579-16 | MF925869 | BOLD:ABV0845 | 658[0n] | 2015 |
| *Solenopsis richteri (A)* | MACN-bar-ins-ct 07001 | ANTPI602-16 | MF925738 | BOLD:ABV0845 | 658[0n] | 2015 |
| *Strumigenys* | T5W42008PNIH05 | ANTPI089-10 |  |  | 0 | 2008 |
| *Strumigenys appretiata* | MACN-Bar-Ins-ct 05155 | ANTPI263-13 |  |  | 0 | 2008 |
| *Strumigenys appretiata* | MACN-bar-ins-ct 06914 | ANTPI515-15 |  |  | 0 | 2008 |
| *Strumigenys crassicornis* | MACN-Bar-Ins-ct 06785 | ANTPI386-15 |  |  | 0 | 1999 |
| *Strumigenys denticulata* | MACN-Bar-Ins-ct 06861 | ANTPI462-15 |  |  | 0 | 1999 |
| *Strumigenys denticulata* | T1W52008PNIH02 | ANTPI086-10 |  |  | 0 | 2008 |
| *Strumigenys denticulata* | T6W32008PNIF11 | ANTPI071-10 |  |  | 0 | 2008 |
| *Strumigenys denticulata* | T6W42008PNIE03 | ANTPI051-10 |  |  | 0 | 2008 |
| *Strumigenys elongata* | MACN-bar-ins-ct 06473 | ANTI176-15 | MF925891 | BOLD:ACZ3541 | 658[0n] | 2015 |
| *Strumigenys elongata* | MACN-bar-ins-ct 06477 | ANTI180-15 | MF925998 | BOLD:ACZ3541 | 658[0n] | 2015 |
| *Strumigenys elongata* | MACN-bar-ins-ct 06940 | ANTPI541-15 | MF925947 | BOLD:ACZ3541 | 658[0n] | 2015 |
| *Strumigenys elongata* | MACN-Bar-Ins-ct 06864 | ANTPI465-15 |  |  | 0 | 1999 |
| *Strumigenys elongata* | MACN-Bar-Ins-ct 05092 | ANTPI234-13 |  |  | 0 | 2008 |
| *Strumigenys louisianae* | MACN-Bar-Ins-ct 06829 | ANTPI430-15 |  |  | 0 | 1999 |
| *Strumigenys louisianae* | T1W32008PNID11 | ANTPI047-10 |  |  | 0 | 2008 |
| *Strumigenys ogloblini* | MACN-bar-ins-ct 06476 | ANTI179-15 | MF925865 | BOLD:ACZ4305 | 658[0n] | 2015 |
| *Strumigenys ogloblini* | MACN-Bar-Ins-ct 06839 | ANTPI440-15 |  |  | 0 | 1999 |
| *Strumigenys PEH01* | MACN-Bar-Ins-ct 06821 | ANTPI422-15 |  |  | 0 | 1999 |
| *Strumigenys PEH01* | MACN-Bar-Ins-ct 06849 | ANTPI450-15 |  |  | 0 | 1999 |
| *Strumigenys PEH01* | MACN-Bar-Ins-ct 06855 | ANTPI456-15 |  |  | 0 | 1999 |
| *Strumigenys PEH02* | MACN-Bar-Ins-ct 06871 | ANTPI472-15 |  |  | 0 | 1999 |
| *Tapinoma atriceps* | MACN-bar-ins-ct 06905 | ANTPI506-15 |  |  | 0 | 1999 |
| *Tapinoma atriceps* | MACN-bar-ins-ct 06910 | ANTPI511-15 |  |  | 0 | 1999 |
| *Trachymyrmex PEH01* | MACN-bar-ins-ct 06953 | ANTPI554-15 |  |  | 0 | 2015 |
| *Wasmannia auropunctata* | MACN-Bar-Ins-ct 05143 | ANTPI252-13 | MF925747 | BOLD:ACH5104 | 658[0n] | 2011 |
| *Wasmannia auropunctata* | MACN-Bar-Ins-ct 05156 | ANTPI264-13 | MF926000 | BOLD:ACH5104 | 658[0n] | 2011 |
| *Wasmannia rochai* | MACN-bar-ins-ct 06907 | ANTPI508-15 | MF925952 | BOLD:ACZ3037 | 658[0n] | 2015 |
| *Wasmannia rochai* | MACN-Bar-Ins-ct 06787 | ANTPI388-15 |  |  | 0 | 1999 |
| *Wasmannia rochai* | MACN-Bar-Ins-ct 06837 | ANTPI438-15 |  |  | 0 | 1999 |
| *Wasmannia rochai* | MACN-Bar-Ins-ct 06858 | ANTPI459-15 |  |  | 0 | 1999 |
| *Wasmannia rochai* | MACN-Bar-Ins-ct 06869 | ANTPI470-15 |  |  | 0 | 1999 |

Table S2. Summary of the 124 species that constituted the dataset used for the analyses. For each species we report the sampling size (N), the mean and maximum intraspecific distances, and the minimum distance to the nearest neighbor (heterospecific). We also show the correspondence between MOTUs and species boundaries for each clustering algorithm (see Materials and methods for more details). Numbers in brackets after the SPLIT category indicate the number of groups in which the species was divided.

| Species (124) | N | Mean distance (% K2P) | Max distance (% K2P) | Min distance to NN | RESL (137) | TCS 95% (136) | ABGD initial partition (125) | ABGD recursive P = 1.29% (132) | ABGD recursive P = 0.28% (136) |
| --- | --- | --- | --- | --- | --- | --- | --- | --- | --- |
| *Acanthostichus brevicornis* | 1 | NA | NA | 8.59 | MATCH | MATCH | MATCH | MATCH | MATCH |
| *Acanthostichus quadratus* | 2 | 0.00 | 0.00 | 8.59 | MATCH | MATCH | MATCH | MATCH | MATCH |
| *Apterostigma PEH01* | 1 | NA | NA | 15.35 | MATCH | MATCH | MATCH | MATCH | MATCH |
| *Apterostigma PEH02* | 2 | 0.15 | 0.15 | 15.35 | MATCH | MATCH | MATCH | MATCH | MATCH |
| *Atta sexdens* | 8 | 1.60 | 2.97 | 19.42 | SPLIT (3) | SPLIT (2) | MATCH | SPLIT (3) | SPLIT (3) |
| *Azteca adrepens* | 3 | 0.20 | 0.30 | 20.52 | MATCH | MATCH | MATCH | MATCH | MATCH |
| *Brachymyrmex antennatus* | 1 | NA | NA | 13.52 | MATCH | MATCH | MATCH | MATCH | MATCH |
| *Brachymyrmex aphidicola* | 2 | 0.00 | 0.00 | 10.44 | MATCH | MATCH | MATCH | MATCH | MATCH |
| *Brachymyrmex cordemoyi* | 4 | 0.00 | 0.00 | 10.44 | MATCH | MATCH | MATCH | MATCH | MATCH |
| *Camponotus atriceps* | 2 | 0.00 | 0.00 | 11.31 | MATCH | MATCH | MATCH | MATCH | MATCH |
| *Camponotus brasiliensis* | 1 | NA | NA | 18.37 | MATCH | MATCH | MATCH | MATCH | MATCH |
| *Camponotus cf. landolti* | 4 | 0.00 | 0.00 | 16.87 | MATCH | MATCH | MATCH | MATCH | MATCH |
| *Camponotus cingulatus* | 6 | 0.05 | 0.15 | 15.54 | MATCH | MATCH | MATCH | MATCH | MATCH |
| *Camponotus crassus* | 5 | 0.46 | 0.78 | 17.31 | MATCH | MATCH | MATCH | MATCH | SPLIT (2) |
| *Camponotus geralensis* | 1 | NA | NA | 17.21 | MATCH | MATCH | MATCH | MATCH | MATCH |
| *Camponotus PEH01* | 1 | NA | NA | 11.31 | MATCH | MATCH | MATCH | MATCH | MATCH |
| *Camponotus renggeri* | 1 | NA | NA | 13.22 | MATCH | MATCH | MATCH | MATCH | MATCH |
| *Camponotus rufipes* | 4 | 1.17 | 2.33 | 13.22 | SPLIT (2) | SPLIT (2) | MATCH | MATCH | MATCH |
| *Camponotus sericeiventris* | 8 | 0.04 | 0.15 | 17.21 | MATCH | MATCH | MATCH | MATCH | MATCH |
| *Camponotus striatus* | 1 | NA | NA | 16.93 | MATCH | MATCH | MATCH | MATCH | MATCH |
| *Carebara brasiliana* | 1 | NA | NA | 20.18 | MATCH | MATCH | MATCH | MATCH | MATCH |
| *Cephalotes eduarduli* | 2 | 0.00 | 0.00 | 17.50 | MATCH | MATCH | MATCH | MATCH | MATCH |
| *Cephalotes minutus* | 1 | NA | NA | 12.45 | MATCH | MATCH | MATCH | MATCH | MATCH |
| *Cephalotes pusillus* | 2 | 0.00 | 0.00 | 12.45 | MATCH | MATCH | MATCH | MATCH | MATCH |
| *Crematogaster corticicola* | 4 | 0.00 | 0.00 | 14.51 | MATCH | MATCH | MATCH | MATCH | MATCH |
| *Crematogaster curvispinosa* | 2 | 0.00 | 0.00 | 17.41 | MATCH | MATCH | MATCH | MATCH | MATCH |
| *Crematogaster lutzi* | 1 | NA | NA | 14.51 | MATCH | MATCH | MATCH | MATCH | MATCH |
| *Crematogaster montezumia* | 3 | 0.00 | 0.00 | 17.43 | MATCH | MATCH | MATCH | MATCH | MATCH |
| *Crematogaster nigropilosa* | 4 | 0.00 | 0.00 | 17.28 | MATCH | MATCH | MATCH | MATCH | MATCH |
| *Crematogaster PEH02* | 2 | 0.00 | 0.00 | 19.71 | MATCH | MATCH | MATCH | MATCH | MATCH |
| *Cylindromyrmex brasiliensis* | 1 | NA | NA | 19.11 | MATCH | MATCH | MATCH | MATCH | MATCH |
| *Cyphomyrmex rimosus* | 1 | NA | NA | 18.37 | MATCH | MATCH | MATCH | MATCH | MATCH |
| *Dinoponera australis* | 7 | 2.08 | 3.64 | 25.16 | SPLIT (2) | SPLIT (2) | MATCH | SPLIT (2) | SPLIT (2) |
| *Dolichoderus bispinosus* | 4 | 0.10 | 0.15 | 24.54 | MATCH | MATCH | MATCH | MATCH | MATCH |
| *Dolichoderus germaini* | 2 | 0.00 | 0.00 | 22.42 | MATCH | MATCH | MATCH | MATCH | MATCH |
| *Dolichoderus lutosus* | 1 | NA | NA | 20.16 | MATCH | MATCH | MATCH | MATCH | MATCH |
| *Dorymyrmex bruneus* | 2 | 0.00 | 0.00 | 18.75 | MATCH | MATCH | MATCH | MATCH | MATCH |
| *Eciton vagans* | 6 | 0.21 | 0.46 | 16.44 | MATCH | MATCH | MATCH | MATCH | MATCH |
| *Ectatomma brunneum* | 1 | NA | NA | 14.95 | MATCH | MATCH | MATCH | MATCH | MATCH |
| *Ectatomma edentatum* | 5 | 7.57 | 18.97 | 14.95 | SPLIT (2) | SPLIT (2) | SPLIT (2) | SPLIT (2) | SPLIT (2) |
| *Gnamptogenys haenschi* | 1 | NA | NA | 17.78 | MATCH | MATCH | MATCH | MATCH | MATCH |
| *Gnamptogenys striatula* | 4 | 0.08 | 0.15 | 17.83 | MATCH | MATCH | MATCH | MATCH | MATCH |
| *Gnamptogenys triangularis* | 2 | 0.00 | 0.00 | 17.83 | MATCH | MATCH | MATCH | MATCH | MATCH |
| *Heteroponera dolo* | 4 | 0.81 | 1.57 | 9.23 | MATCH | MATCH | MATCH | SPLIT (2) | SPLIT (2) |
| *Heteroponera mayri* | 1 | NA | NA | 9.23 | MATCH | MATCH | MATCH | MATCH | MATCH |
| *Heteroponera microps* | 2 | 0.00 | 0.00 | 14.71 | MATCH | MATCH | MATCH | MATCH | MATCH |
| *Hylomyrma balzani* | 1 | NA | NA | 23.59 | MATCH | MATCH | MATCH | MATCH | MATCH |
| *Hypoponera agilis* | 1 | NA | NA | 14.10 | MATCH | MATCH | MATCH | MATCH | MATCH |
| *Hypoponera cf. opacior* | 2 | 0.30 | 0.30 | 12.98 | MATCH | MATCH | MATCH | MATCH | MATCH |
| *Hypoponera distinguenda* | 2 | 0.00 | 0.00 | 13.01 | MATCH | MATCH | MATCH | MATCH | MATCH |
| *Hypoponera foreli* | 5 | 4.48 | 11.20 | 13.90 | SPLIT (2) | SPLIT (2) | SPLIT (2) | SPLIT (2) | SPLIT (2) |
| *Hypoponera parva* | 2 | 0.00 | 0.00 | 15.20 | MATCH | MATCH | MATCH | MATCH | MATCH |
| *Hypoponera PEH01* | 1 | NA | NA | 15.54 | MATCH | MATCH | MATCH | MATCH | MATCH |
| *Hypoponera PEH02* | 4 | 0.00 | 0.00 | 12.35 | MATCH | MATCH | MATCH | MATCH | MATCH |
| *Hypoponera trigona* | 5 | 6.41 | 9.92 | 12.35 | SPLIT (3) | SPLIT (3) | SPLIT (3) | SPLIT (3) | SPLIT (3) |
| *Labidus coecus* | 8 | 2.32 | 8.12 | 16.44 | SPLIT (2) | SPLIT (2) | SPLIT (2) | SPLIT (2) | SPLIT (4) |
| *Labidus praedator* | 1 | NA | NA | 17.04 | MATCH | MATCH | MATCH | MATCH | MATCH |
| *Leptogenys iheringi* | 1 | NA | NA | 17.95 | MATCH | MATCH | MATCH | MATCH | MATCH |
| *Linepithema iniquum* | 1 | NA | NA | 12.87 | MATCH | MATCH | MATCH | MATCH | MATCH |
| *Linepithema micans* | 4 | 0.08 | 0.15 | 12.87 | MATCH | MATCH | MATCH | MATCH | MATCH |
| *Linepithema pulex* | 1 | NA | NA | 13.05 | MATCH | MATCH | MATCH | MATCH | MATCH |
| *Megalomyrmex brandaoi* | 1 | NA | NA | 14.06 | MATCH | MATCH | MATCH | MATCH | MATCH |
| *Megalomyrmex megadrifti* | 1 | NA | NA | 14.06 | MATCH | MATCH | MATCH | MATCH | MATCH |
| *Megalomyrmex miri* | 1 | NA | NA | 17.78 | MATCH | MATCH | MATCH | MATCH | MATCH |
| *Mycetarotes parallelus* | 1 | NA | NA | 20.50 | MATCH | MATCH | MATCH | MATCH | MATCH |
| *Mycocepurus smithii* | 3 | 0.00 | 0.00 | 25.52 | MATCH | MATCH | MATCH | MATCH | MATCH |
| *Myrmelachista catharinae* | 2 | 0.00 | 0.00 | 14.45 | MATCH | MATCH | MATCH | MATCH | MATCH |
| *Myrmelachista nodigera* | 1 | NA | NA | 14.45 | MATCH | MATCH | MATCH | MATCH | MATCH |
| *Neivamyrmex angustinodis* | 1 | NA | NA | 21.67 | MATCH | MATCH | MATCH | MATCH | MATCH |
| *Neivamyrmex punctaticeps* | 5 | 0.00 | 0.00 | 21.67 | MATCH | MATCH | MATCH | MATCH | MATCH |
| *Neoponera bactronica* | 1 | NA | NA | 0.00 | MERGE | MERGE | MERGE | MERGE | MERGE |
| *Neoponera crenata* | 3 | 3.26 | 4.88 | 4.39 | SPLIT (2) | SPLIT (2) | MERGE | MERGE | MERGE |
| *Neoponera curvinodis* | 1 | NA | NA | 0.00 | MERGE | MERGE | MERGE | MERGE | MERGE |
| *Neoponera fiebrigi* | 1 | NA | NA | 3.92 | MATCH | MATCH | MERGE | MERGE | MERGE |
| *Neoponera moesta* | 2 | 0.92 | 0.92 | 3.92 | MATCH | MATCH | MERGE | MERGE | MERGE |
| *Neoponera obscuricornis* | 3 | 0.00 | 0.00 | 11.10 | MATCH | MATCH | MATCH | MATCH | MATCH |
| *Neoponera verenae* | 1 | NA | NA | 11.10 | MATCH | MATCH | MATCH | MATCH | MATCH |
| *Neoponera villosa* | 3 | 0.20 | 0.30 | 9.98 | MATCH | MATCH | MATCH | MATCH | MATCH |
| *Nesomyrmex asper* | 3 | 0.00 | 0.00 | 19.89 | MATCH | MATCH | MATCH | MATCH | MATCH |
| *Nesomyrmex tonsuratus* | 1 | NA | NA | 20.95 | MATCH | MATCH | MATCH | MATCH | MATCH |
| *Nylanderia fulva* | 1 | NA | NA | 8.26 | MATCH | MATCH | MATCH | MATCH | MATCH |
| *Nylanderia PEH01* | 1 | NA | NA | 8.26 | MATCH | MATCH | MATCH | MATCH | MATCH |
| *Nylanderia PEH02* | 2 | 0.00 | 0.00 | 11.12 | MATCH | MATCH | MATCH | MATCH | MATCH |
| *Octostruma iheringi* | 1 | NA | NA | 20.41 | MATCH | MATCH | MATCH | MATCH | MATCH |
| *Octostruma PEH01* | 1 | NA | NA | 21.20 | MATCH | MATCH | MATCH | MATCH | MATCH |
| *Odontomachus chelifer* | 5 | 0.00 | 0.00 | 9.55 | MATCH | MATCH | MATCH | MATCH | MATCH |
| *Odontomachus meinerti* | 10 | 1.96 | 5.53 | 9.55 | SPLIT (2) | SPLIT (2) | MATCH | SPLIT (2) | SPLIT (2) |
| *Pachycondyla harpax* | 1 | NA | NA | 12.82 | MATCH | MATCH | MATCH | MATCH | MATCH |
| *Pachycondyla PEH01* | 1 | NA | NA | 12.82 | MATCH | MATCH | MATCH | MATCH | MATCH |
| *Pachycondyla striata* | 4 | 0.15 | 0.31 | 15.41 | MATCH | MATCH | MATCH | MATCH | MATCH |
| *Pheidole alpinensis* | 1 | NA | NA | 17.90 | MATCH | MATCH | MATCH | MATCH | MATCH |
| *Pheidole fimbriata* | 3 | 7.06 | 10.59 | 20.19 | SPLIT (2) | SPLIT (2) | SPLIT (2) | SPLIT (2) | SPLIT (2) |
| *Pheidole mosenopsis* | 2 | 0.30 | 0.30 | 17.91 | MATCH | MATCH | MATCH | MATCH | MATCH |
| *Pheidole obscurithorax* | 1 | NA | NA | 15.12 | MATCH | MATCH | MATCH | MATCH | MATCH |
| *Pheidole PEH01* | 7 | 0.07 | 0.15 | 16.50 | MATCH | MATCH | MATCH | MATCH | MATCH |
| *Pheidole PEH02* | 7 | 2.75 | 5.91 | 12.52 | SPLIT (2) | SPLIT (2) | MATCH | SPLIT (2) | SPLIT (2) |
| *Pheidole PEH03* | 3 | 0.10 | 0.15 | 16.64 | MATCH | MATCH | MATCH | MATCH | MATCH |
| *Pheidole PEH04* | 4 | 0.00 | 0.00 | 16.09 | MATCH | MATCH | MATCH | MATCH | MATCH |
| *Pheidole PEH05* | 1 | NA | NA | 16.09 | MATCH | MATCH | MATCH | MATCH | MATCH |
| *Pheidole PEH06* | 1 | NA | NA | 15.12 | MATCH | MATCH | MATCH | MATCH | MATCH |
| *Pheidole PEH09* | 2 | 0.00 | 0.00 | 12.52 | MATCH | MATCH | MATCH | MATCH | MATCH |
| *Pheidole rugatula* | 4 | 0.00 | 0.00 | 18.67 | MATCH | MATCH | MATCH | MATCH | MATCH |
| *Pheidole sigillata* | 5 | 0.24 | 0.61 | 18.68 | MATCH | MATCH | MATCH | MATCH | MATCH |
| *Pheidole subarmata* | 15 | 1.09 | 2.19 | 17.99 | SPLIT (2) | SPLIT (2) | MATCH | SPLIT (2) | SPLIT (2) |
| *Platythyrea pilosula* | 1 | NA | NA | 22.18 | MATCH | MATCH | MATCH | MATCH | MATCH |
| *Pogonomyrmex naegelii* | 1 | NA | NA | 21.18 | MATCH | MATCH | MATCH | MATCH | MATCH |
| *Procryptocerus adlerzi* | 1 | NA | NA | 16.17 | MATCH | MATCH | MATCH | MATCH | MATCH |
| *Procryptocerus hylaeus* | 3 | 0.20 | 0.30 | 17.91 | MATCH | MATCH | MATCH | MATCH | MATCH |
| *Procryptocerus regularis* | 1 | NA | NA | 16.17 | MATCH | MATCH | MATCH | MATCH | MATCH |
| *Pseudomyrmex gracilis* | 1 | NA | NA | 5.84 | MATCH | MATCH | MERGE | MERGE | MERGE |
| *Pseudomyrmex PEH01* | 1 | NA | NA | 16.21 | MATCH | MATCH | MATCH | MATCH | MATCH |
| *Pseudomyrmex PEH02* | 1 | NA | NA | 5.84 | MATCH | MATCH | MERGE | MERGE | MERGE |
| *Pseudomyrmex schuppi* | 1 | NA | NA | 21.60 | MATCH | MATCH | MATCH | MATCH | MATCH |
| *Pseudomyrmex simplex* | 1 | NA | NA | 16.21 | MATCH | MATCH | MATCH | MATCH | MATCH |
| *Solenopsis helena* | 2 | 0.00 | 0.00 | 17.10 | MATCH | MATCH | MATCH | MATCH | MATCH |
| *Solenopsis iheringi* | 1 | NA | NA | 19.49 | MATCH | MATCH | MATCH | MATCH | MATCH |
| *Solenopsis PEH01* | 1 | NA | NA | 4.67 | MATCH | MATCH | MERGE | MERGE | MERGE |
| *Solenopsis PEH02* | 1 | NA | NA | 18.86 | MATCH | MATCH | MATCH | MATCH | MATCH |
| *Solenopsis PEH06* | 2 | 0.00 | 0.00 | 4.67 | MATCH | MATCH | MERGE | MERGE | MERGE |
| *Solenopsis richteri* | 5 | 0.00 | 0.00 | 17.10 | MATCH | MATCH | MATCH | MATCH | MATCH |
| *Strumigenys elongata* | 3 | 0.20 | 0.30 | 17.45 | MATCH | MATCH | MATCH | MATCH | MATCH |
| *Strumigenys ogloblini* | 1 | NA | NA | 17.45 | MATCH | MATCH | MATCH | MATCH | MATCH |
| *Wasmannia auropunctata* | 2 | 0.00 | 0.00 | 19.38 | MATCH | MATCH | MATCH | MATCH | MATCH |
| *Wasmannia rochai* | 1 | NA | NA | 19.53 | MATCH | MATCH | MATCH | MATCH | MATCH |

**Table S3.** Results of the TCS analyses for a range of ten parsimony limit (i.e. cut-off) values.

| Parsimony probability | Maximum connection steps | Subnetworks |
| --- | --- | --- |
| 90% | 16 | 133 |
| 91% | 15 | 134 |
| 92% | 14 | 135 |
| 93% | 13 | 135 |
| 94% | 12 | 136 |
| 95% | 11 | 136 |
| 96% | 9 | 136 |
| 97% | 8 | 138 |
| 98% | 6 | 138 |
| 99% | 4 | 140 |

**Table S4.** Results of the ABGD analyses for two distances metrics (p-distance and K2P), two relative gap values (X = 0.8 and X = 1) and a range of prior intraspecific divergence (P) values between 0.1% and 10%.

|  |  |  | **Prior intraspecific divergence (P, %)** | | | | | | | | | |
| --- | --- | --- | --- | --- | --- | --- | --- | --- | --- | --- | --- | --- |
| **Distance model** | X | Partition | 0.1 | 0.17 | 0.28 | 0.46 | 0.77 | 1.29 | 2.15 | 3.59 | 5.99 | 10 |
| **p-distance** | 1 | Initial | 125 | 125 | 125 | 125 | 125 | 125 | 125 | 125 | 125 | 1 |
|  |  | Recursive | 147 | 135 | 135 | 135 | 132 | 132 | 128 | 127 | 125 | 1 |
|  |  |  |  |  |  |  |  |  |  |  |  |  |
|  | 0.8 | Initial | 125 | 125 | 125 | 125 | 125 | 125 | 125 | 125 | 125 | 1 |
|  |  | Recursive | 157 | 140 | 140 | 139 | 136 | 136 | 132 | 130 | 125 |  |
| **K2P** | 1 | Initial | 125 | 125 | 125 | 125 | 125 | 125 | 125 | 125 | 125 | 1 |
|  |  | Recursive | 147 | 135 | 135 | 135 | 132 | 132 | 128 | 128 | 125 | 1 |
|  |  |  |  |  |  |  |  |  |  |  |  |  |
|  | 0.8 | Initial | 125 | 125 | 125 | 125 | 125 | 125 | 125 | 125 | 125 | 1 |
|  |  | Recursive | 157 | 140 | 140 | 139 | 136 | 136 | 132 | 131 | 125 | 1 |

**Figure Legends**

Fig. S1 Histogram of threshold optimization method reporting the frequencies of false-positive and false-negative identifications across thresholds values from 0.1% to 10%.

Fig. S2 Density plot of genetic distances generated by the function ‘localMinima’ in SPIDER.

Fig. S3 Morphological differences between the two MOTUs of *Ectatomma edentatum* (left: MACN-Bar-Ins-ct 5123, right: MACN-Bar- Ins-ct 6433).

Fig. S4 Subpetiolar process of male MACN-Bar-Ins-ct 02564 identified using our library as *N. crenata*.

Fig S1


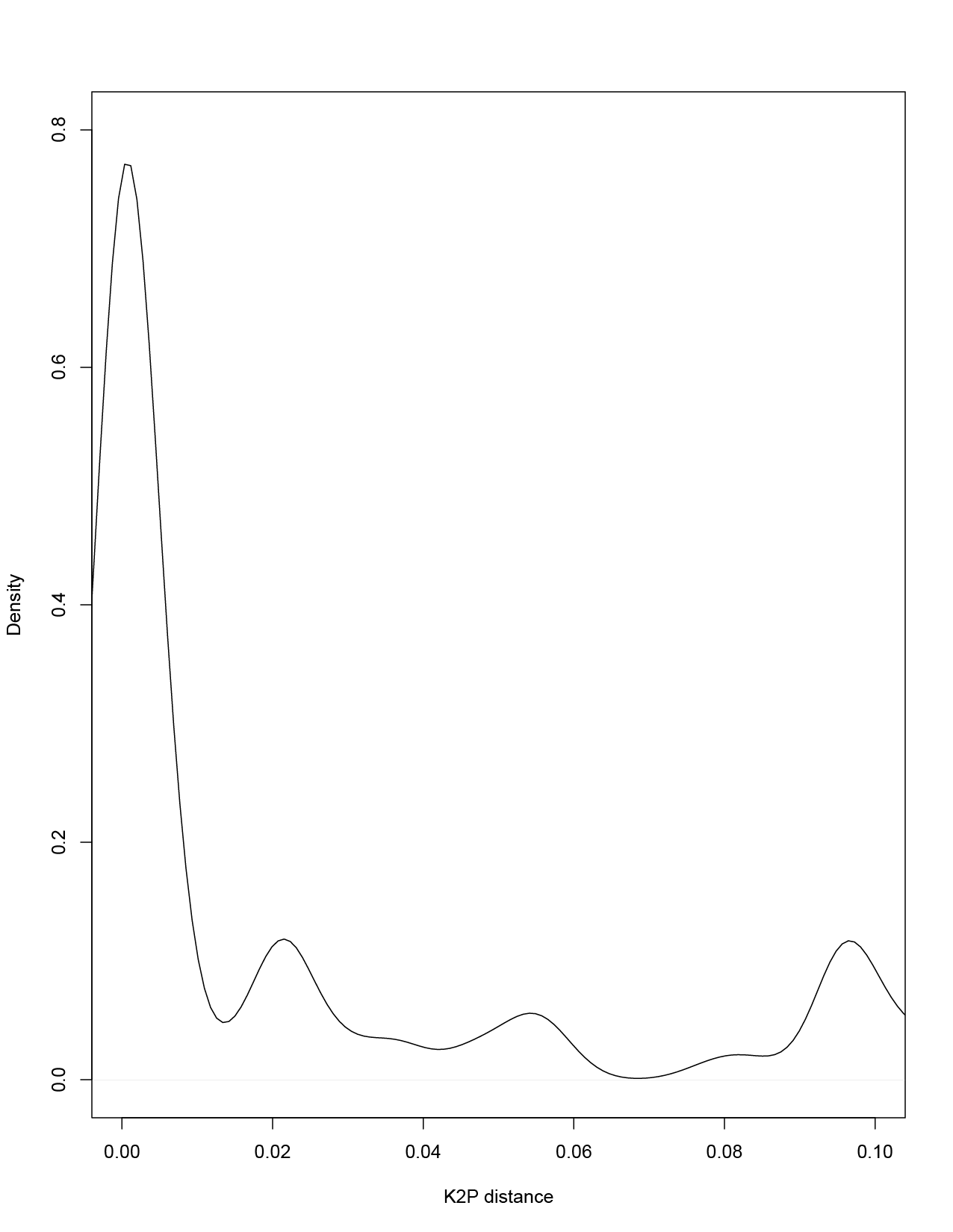


Fig S2


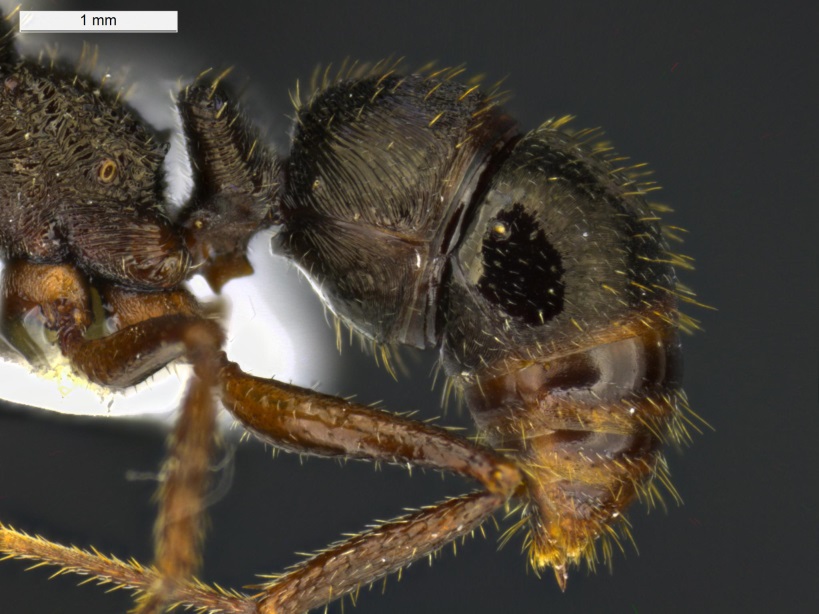

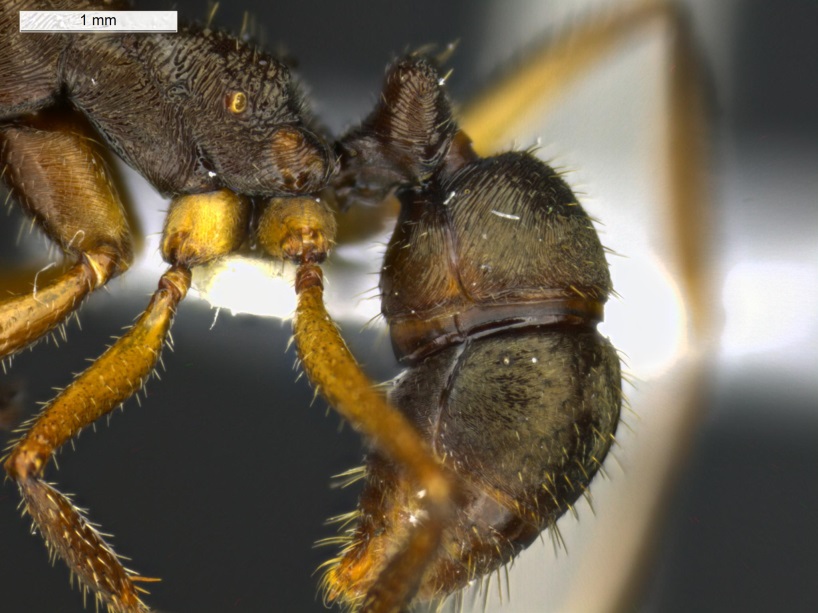
Fig S3


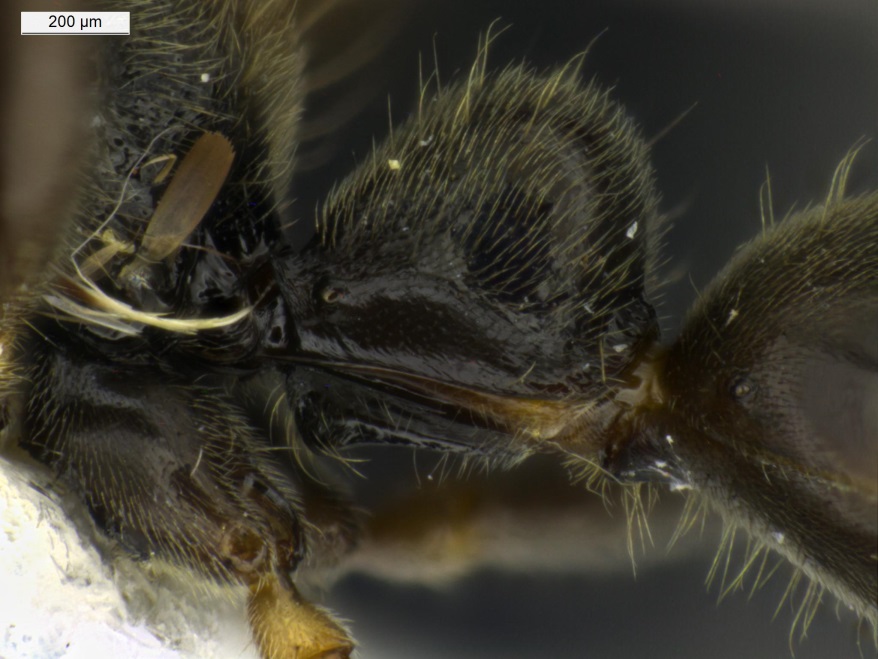


Fig. S4
